# Vitamin C‐Dependent Intergenerational Inheritance of Enhanced Endurance Performance Following Maternal Exercise

**DOI:** 10.1002/advs.202408912

**Published:** 2025-02-08

**Authors:** Haiwang Shi, Jie Li, Fan Li, Haoyang Yu, Fulong Zhang, Tao Wu, Luodan Yang, Yuecheng Li, Rui Hu, Mengjie Chen, Nina SG, Xuhong Zhuang, Shu Feng, Ling Zhu, Rui Duan

**Affiliations:** ^1^ School of Physical Education and Sports Science South China Normal University Guangzhou Guangdong 510006 China

**Keywords:** epigenetics, exercise, maternal exercise, offspring health, skeletal muscle

## Abstract

Declining levels of physical activity and fitness in children and youth are linked to negative health outcomes. This study investigates whether maternal exercise can enhance offspring's physical fitness. Our results demonstrate that maternal exercise improves offspring's endurance by changing muscle fiber composition and promoting mitochondrial biogenesis, with benefits lasting across generations. This improvement is associated with changes in DNA methylation, specifically the demethylation of the *Slc23a2* gene, which codes for SVCT2, crucial for vitamin C (VC) transport, in F1 and F2 generations. Importantly, VC administration during pregnancy mimics the transgenerational benefits of exercise on offspring fitness, but these benefits are absent in genetic VC deficiency mice. VC supplementation increases TET2 expression in murine and human myogenic cells, regulating DNA methylation, promoting the development of oxidative fibers, and enhancing mitochondrial biogenesis. This study highlights the VC‐TET2‐SVCT2 pathway as a key mechanism for the transgenerational endurance benefits of maternal exercise, suggesting potential strategies to enhance maternal and child health.

## Graphical abstract

This article demonstrates that maternal exercise or vitamin C supplementation during pregnancy enhance offspring muscle function by promoting mitochondrial biogenesis and oxidative fiber formation. This epigenetically driven improvement ultimately leads to higher endurance performance, strongly suggesting that sufficient maternal physical activity can influence skeletal muscle health and fitness across future generations.

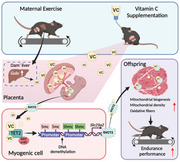

## 1 Introduction

Physical fitness is a multidimensional construct encompassing elements such as muscle strength, muscular endurance, speed, flexibility, balance, and coordination.^[^ ^1^, ^2^ ^]^ Sufficient levels of physical fitness are robustly correlated with decreased risks of all‐cause mortality, cardiovascular disease, insulin resistance, and metabolic disorders.^[^ ^3^, ^4^, ^5^, ^6^ ^]^ However, there is growing concern over the global decline in physical fitness levels, particularly among children, with notable decreases in cardiorespiratory and muscular fitness.^[^ ^7^, ^8^, ^9^, ^10^ ^]^ The development of physical fitness is influenced by both genetic factors and regular exercise.^[^ ^11^ ^]^ While DNA provides the foundational blueprint for physical capabilities, gene expression can be significantly modulated by environmental factors and lifestyle choices,^[^ ^12^ ^]^ including parental physical activity.^[^ ^13^ ^]^ Recent studies have underscored the profound impact of parental, particularly maternal, exercise on offspring health outcomes.^[^ ^14^, ^15^, ^16^ ^]^ However, the specific effects of maternal exercise on offspring physical fitness remain underexplored. In this study, we demonstrate that maternal exercise enhances endurance performance in offspring by increasing the proportion of oxidative myofibers and promoting mitochondrial biogenesis in their skeletal muscles. Remarkably, these endurance improvements can be intergenerationally transmitted, suggesting a heritable mechanism beyond direct genetic inheritance.

Given that maternal exercise cannot alter the offspring's DNA sequence in general, we speculate that epigenetic modifications mediate the observed enhancements in endurance performance. Epigenetic alterations, including DNA methylation, histone modifications, and microRNA regulation during embryological development, have been proposed to influence offspring metabolism.^[^ ^17^ ^]^ Changes in DNA methylation play a pivotal role in the benefits of maternal exercise on offspring, such as enhanced DNA demethylation of the *Prdm16* promoter, which promotes brown adipose tissue development and prevents obesity in offspring challenged with a high‐energy diet.^[^ ^13^ ^]^ In addition, maternal exercise protects the fetus from hypermethylation of *Ppargc1α* in skeletal muscle induced by a maternal high‐fat diet.^[^ ^18^ ^]^ These findings highlight the significance of epigenetic mechanisms in translating maternal exercise into tangible health benefits for offspring.

Vitamin C (VC), also known as ascorbic acid, is an essential micronutrient pivotal for various biological functions, including antioxidant activity, regulation of HIF‐1 hydroxylation, metabolism, and collagen synthesis.^[^ ^19^, ^20^, ^21^ ^]^ Recent research has demonstrated that VC can stimulate the enzymatic function of ferrous iron‐ and α‐ketoglutarate‐dependent dioxygenase (αKGDDs), including Jumonji C domain‐containing histone demethylases (JmjC KDMs) and ten‐eleven translocation methylcytosine dioxygenases (TETs).^[^ ^22^, ^23^, ^24^, ^25^ ^]^ These epigenetic hydroxylases facilitate the conversion of 5‐methyl‐cytosine (5mC) to 5‐hydroxy‐methyl‐cytosine (5hmC), a process that generally supports chromatin opening and activation of gene expression.^[^ ^26^, ^27^ ^]^

To identify novel epigenetic targets through which maternal exercise may enhance offspring endurance performance, we perform methylation‐capture sequencing (MCC‐Seq) on skeletal muscle from F1 and F2 offspring. Our analysis reveals significant decreases in DNA methylation of the *Slc23a2* gene across both male and female F1 and F2 offspring. The *Slc23a2* gene encodes the SVCT2 protein, a sodium‐dependent VC transporter responsible for transporting VC from blood to tissues.^[^ ^28^ ^]^ Given the role of VC in epigenetic regulation and the observed demethylation of the *Slc23a2* gene in our MCC‐Seq analysis, we hypothesize that VC and its transporter SVCT2 are key mediators in the epigenetic modifications induced by maternal exercise, thereby enhancing offspring endurance performance.

In this study, we investigate whether VC supplementation could replicate the beneficial effects of maternal exercise on offspring endurance. Our findings demonstrate that exogenous VC supplementation during gestation mimics the effects of maternal exercise, enhancing endurance performance in offspring. In contrast, disrupting VC synthesis by knocking out the *L‐gulonolactone oxidase* (*Gluo*) gene abolishes these beneficial effects. Further *ex vivo* studies show that VC supplementation promotes oxidative myofiber generation and mitochondrial biogenesis in both murine and human myogenic cells. Importantly, *Tet2* knock‐down eliminates these effects, suggesting that maternal exercise improves offspring endurance performance through VC/TET2‐induced DNA methylation changes. These results indicate that maternal exogenous VC supplementation may serve as a novel intervention to mimic maternal exercise, offering a potential strategy to improve offspring physical fitness.

## 2 Results

### 2.1 Maternal Exercise Exerts Intergenerational Effects on the Endurance Performance of the Offspring

**Figure 1:**
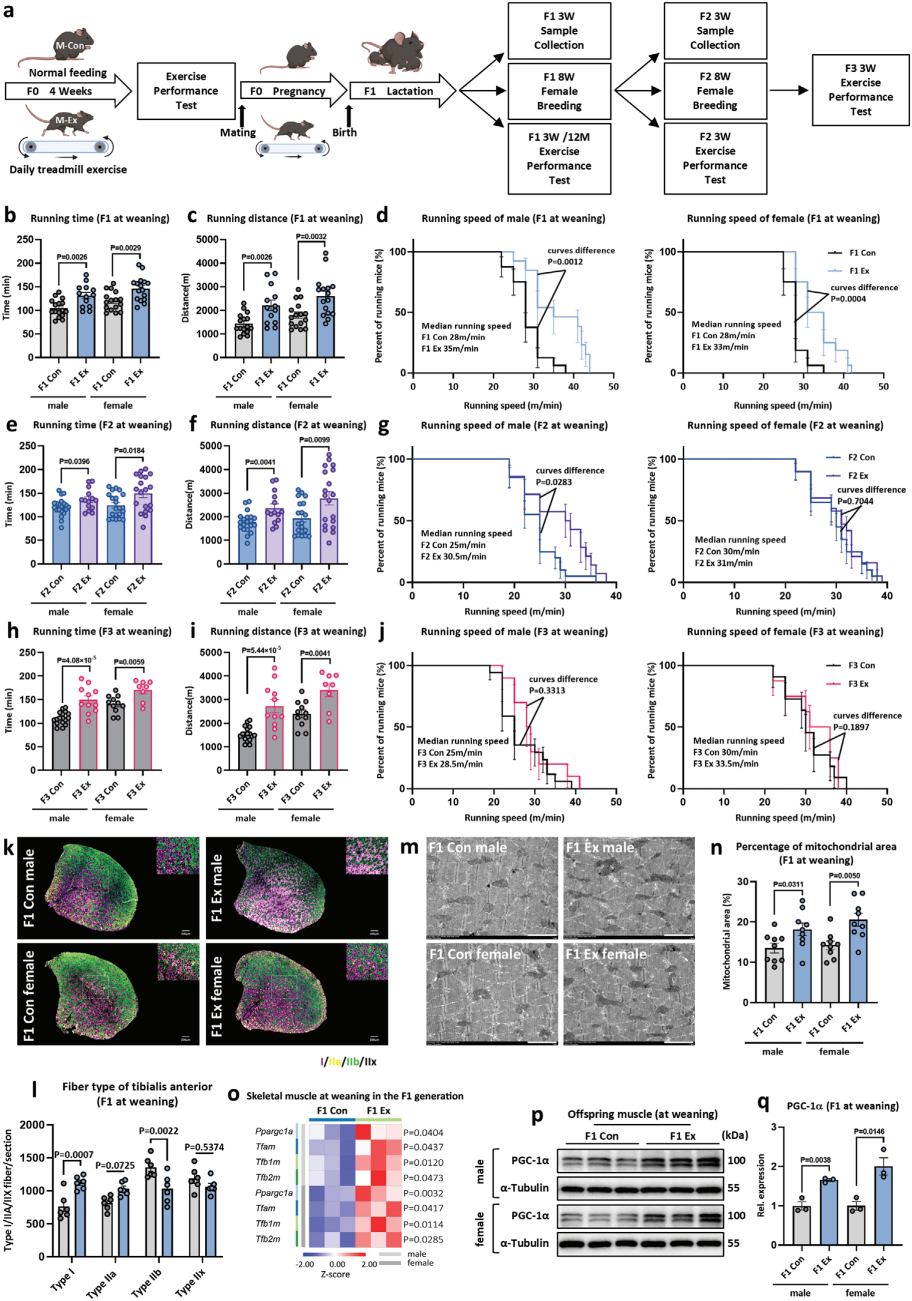
Intergenerational genetic effects and physiological basis of maternal exercise improving endurance performance in offspring. a) A schematic diagram. Female C57BL/6J mice were randomly divided into the control group (M‐Con) and the exercise group (M‐Ex). Mice in the M‐Ex group underwent 4 weeks of treadmill exercise, while the M‐Con group remained sedentary, followed by an endurance exercise performance test. After the test, the animals were mated with age‐matched wild‐type male mice (10‐week‐old) to obtain F1 generation, among which the M‐Ex group received reduced‐load treadmill training during pregnancy until two to three days before delivery. F1 generation mice underwent exercise performance tests at weaning and then mated with age‐matched wild male mice at 8 weeks of age to obtain F2 generation. The same procedure was used to obtain F3 generation. b–j) Endurance performance tests at weaning of the F1, F2, and F3 generations. Running time (b,e,h), distance (c,f,i), and population were plotted against running speed to exhaustion (d,g,j) (Male: F1 Con *n* = 16, F1 Ex *n* = 13, F2 Con *n* = 20, F2 Ex *n* = 14, F3 Con *n* = 17, and F3 Ex *n* = 11. Female: F1 Con *n* = 15, F1 Ex *n* = 16, F2 Con *n* = 20, F2 Ex *n* = 19, F3 Con *n* = 11, and F3 Ex *n* = 8). k,l) Representative immunofluorescence staining for MyHC‐I, MyHC‐IIa, and MyHC‐IIb (k, the scale bar represents 200 µm) and fiber type composition (l) in skeletal muscles in F1 generation (*n* = 6). m,n) Representative TEM micrographs of skeletal muscle showing IMF mitochondria in sections from F1 generation (m, the scale bar represents 2 µm), and the percentage of mitochondrial area per muscle fiber area was quantified from the electron micrographs (n) (*n* = 3, three random statistical electron micrographs of each sample). o) Heatmap visualizing expression of genes related to mitochondrial biogenesis in F1 generation at weaning, detected by RT‐qPCR (*n* = 3). p,q) Representative images of Western blot analysis of PGC‐1α in F1 generation skeletal muscle at weaning (*n* = 3). Statistical analyses were performed using *t*‐test (b,c,e,f,h,i,l,n,o,q) and Log‐rank test (d,g,j). Data are presented as mean ± SEM.

To elucidate the impact of maternal exercise on muscular endurance, muscular strength, and running speed of offspring, prepuberal female mice (5‐week‐old, F0 generation) were randomly assigned to either a maternal sedentary control group (M‐Con) or a maternal exercised group (M‐Ex) (**Figure** **1**a). The M‐Ex group underwent a treadmill training regimend, while the M‐Con group maintained a normal lifestyle. Following a 4‐week treadmill exercise protocol, exercised mothers exhibited significant increases in running time to exhaustion, total distance, and maximum running speed by 15.5%, 32.5%, and 17.1%, respectively (Figure S1a–c, Supporting Information). Subsequent to the exercise performance test, Female F0 mice (10‐week‐old) were mated with age‐matched sedentary wild‐type male mice to produce F1 offspring (Figure 1a). At weaning, F1 offspring from the M‐Ex group demonstrated enhanced exercise performance, evidenced by increased running time to exhaustion, total distance, and maximal running speed (Figure 1b–d). No significant differences were observed in muscle strength, as measured by relative forelimb grip strength (Figure S1d, Supporting Information). In addition, the physical activity propensity of F1 mice was assessed using a freewheel assay. F1 offspring from M‐Ex group exhibited a significant increase in running distance on the 6^th^ and 7^th^ days of testing (Figure S1e, Supporting Information). Importantly, the positive influence of maternal exercise on offspring physical fitness could still be observed at the age of 12 months (Figure S1f–i, Supporting Information).

To investigate the transgenerational effects of maternal exercise, female F1 offspring (10‐week‐old) were mated with age‐matched wild‐type male mice to produce the F2 offspring (Figure 1a). Similar enhancements in endurance performance were detected in both male and female offspring from exercised grandmothers, as marked by increased running duration and total distance (Figure 1e,f). An increase in maximal running speed was consistently observed in F2 male offspring, whereas this effect was not present in female F2 offspring (Figure 1g). Moreover, physical activity propensity, characterized by increased velocity and distance moved in an open field, was also observed in F2 offspring (Figure S1j–l, Supporting Information). Interestingly, the endurance performance of F3 generation mice derived from exercised great‐grandmothers remained notably higher than that of control offspring (Figure 1h,i), while the improvements in maximal running speed were no longer observed (Figure 1j). Same as F1 generation, the forelimb grip strength of F2 and F3 did not show significant difference between offspring of exercised and sedentary dams (Figure S1m,n, Supporting Information).

To rule out strain‐specific effects, the experiment was replicated using Kunming mice, an outbred strain. Consistent with results obtained in C57BL/6J mice, F1 and F2 generation offspring from exercised F0 Kunming mothers showed comparable enhancements in endurance performance following maternal exercise training (Figure S2, Supporting Information). Collectively, these findings demonstrate that maternal exercise enhances offspring physical fitness, with improved endurance performance in the F1 generation being transmitted across multiple generations. Maternal exercise may also have intergenerational effects on the maximum running speed of mice, although this effect does not persist as long as the enhancement in endurance performance.

### 2.2 Maternal Exercise Alters Offspring's Skeletal Muscle Fiber Type Composition and Enhances Mitochondrial Biogenesis

In general, exercise performance is closely associated with skeletal muscle characteristics, including myofiber cross‐sectional area (CSA) and myofiber‐type composition.^[^ ^29^, ^30^ ^]^ To assess the impact of maternal exercise on these parameters, immunofluorescence staining of myosin heavy chain (MyHC) isoforms was performed on the skeletal muscles of F1 offspring. Maternal exercise significantly increased the proportion of oxidative fibers (type I and type IIa myofibers), while reducing the proportion of glycolytic fibers (type IIb myofibers) in the F1 skeletal muscle (Figure 1k,l). These findings were corroborated by quantitative PCR analysis, which revealed elevated expression levels of *Myh7* and *Myh2*‐markers of type I and type IIa myofibers, respectively, and decreased expression of *Myh4* (type IIb) and *Myh1* (type IIx) in F1 skeletal muscle (Figure S3a, Supporting Information). Collectively, these data suggest that maternal exercise modulates myofiber‐type composition in F1 offspring, thereby contributing to enhanced endurance performance. Further, maternal exercise markedly increased the mean fiber CSA of the skeletal muscle in F1 offspring (Figure S3b,c, Supporting Information). Specifically, the average fiber CSA was elevated by 41.9% in male offspring and by 24.1% in female offspring (Figure S3c, Supporting Information). The frequency distribution of the CSA in the total muscle fiber population of F1 mice shifted to the right, indicating an overall enlargement of muscle fibers in offspring from exercised dams (Figure S3d, Supporting Information).

An increased mitochondrial quantity in skeletal muscle is essential for enhancing endurance performance and exercise capacity.^[^ ^31^, ^32^ ^]^ To evaluate mitochondrial biogenesis, transmission electron microscopy (TEM) was utilized to measure mitochondrial volume density within the intermyofibrillar (IMF) compartments of F1 skeletal muscle at weaning. TEM analysis revealed a significant increase in mitochondrial volume density in the F1 Ex group compared to the F1 Con group (Figure 1m,n). In addition, co‐staining of the mitochondrial membrane protein marker TOM20 with specific MyHC isoforms revealed that maternal exercise selectively increased the number of TOM20‐positive fibers in the offspring's skeletal muscle (Figure S4, Supporting Information). In male offspring, the proportion of TOM20‐positive type I and type IIa fibers was significantly higher compared to controls, whereas type IIb and type IIx fibers showed no changes. In female offspring, only the proportion of TOM20‐positive type I fibers increased, with no statistical differences observed in type IIa, IIb, or IIx fibers. However, immunofluorescence data cannot determine single‐fiber mitochondrial content changes within each oxidative fiber; instead, these findings indicate that maternal exercise elevates the fraction of oxidative fibers displaying robust TOM20 signals, without affecting the number of TOM20‐positive fibers in glycolytic fiber types. In line with these data, mitochondrial DNA (mtDNA) copy number and the expression of genes involved in mitochondrial biogenesis, such as *Ppargc1a*, *Tfam*, *Tfb1m*, and *Tfb2m*, were assessed. Maternal exercise significantly upregulated the expression of these mitochondrial biogenesis‐related genes and increased mtDNA content in both female and male F1 offspring at weaning (Figure 1o; Figure S3e, Supporting Information). Moreover, protein levels of PGC‐1α, a key regulator of mitochondrial biogenesis, were also enhanced in F1 offspring (Figure 1p,q).

Notably, the expression levels of PGC‐1α and mitochondrial biogenesis‐related genes were also elevated in the skeletal muscle of F2 generation offspring (Figure S5a–f, Supporting Information). Concurrently, succinate dehydrogenase (SDH) staining revealed a significantly higher number of SDH‐positive fibers (indicated by dark staining) in the F2 Ex group compared to the F2 Con group (Figure S5g,h, Supporting Information). SDH is a mitochondrial enzyme that plays a vital role in the tricarboxylic acid cycle, catalyzing the oxidation of succinate to fumarate and transferring electrons from FADH to CoQ.^[^ ^33^ ^]^ Regions displaying higher levels of SDH activity and staining indicate a greater abundance of mitochondria and enhanced mitochondrial function. However, no significant differences were observed between the F2 Ex and F2 Con group in terms of average CSA and fiber frequency domain distribution (Figure S5i,j, Supporting Information). In summary, maternal exercise in the F0 generation leads to an increased proportion of type I and type IIa myofibers and enhances mitochondrial number and function in the offspring's skeletal muscle, thereby contributing to improved endurance performance across multiple generations.

Interestingly, maternal exercise significantly inhibited the accumulation of abdominal fat and attenuated the increase in body weight in F1 offspring (Figure S3f–h, Supporting Information). However, skeletal muscle mass was not significantly affected at weaning or at 12 months of age (Figure S3i,j, Supporting Information). These findings may be explained by Speakman's Heat Dissipation Limit theory^[^ ^34^ ^]^ which posits that organisms have a finite capacity to dissipate metabolic heat. When maternal exercise raises overall energy expenditure, the system nears its thermal limit. Consequently, more energy is diverted toward heat dissipation and less is allocated to adipose storage, thereby restricting fat accumulation and overall body weight gain in developing offspring.

### 2.3 Maternal Exercise Reprograms DNA Methylation in the Offspring's Skeletal Muscle

**Figure 2:**
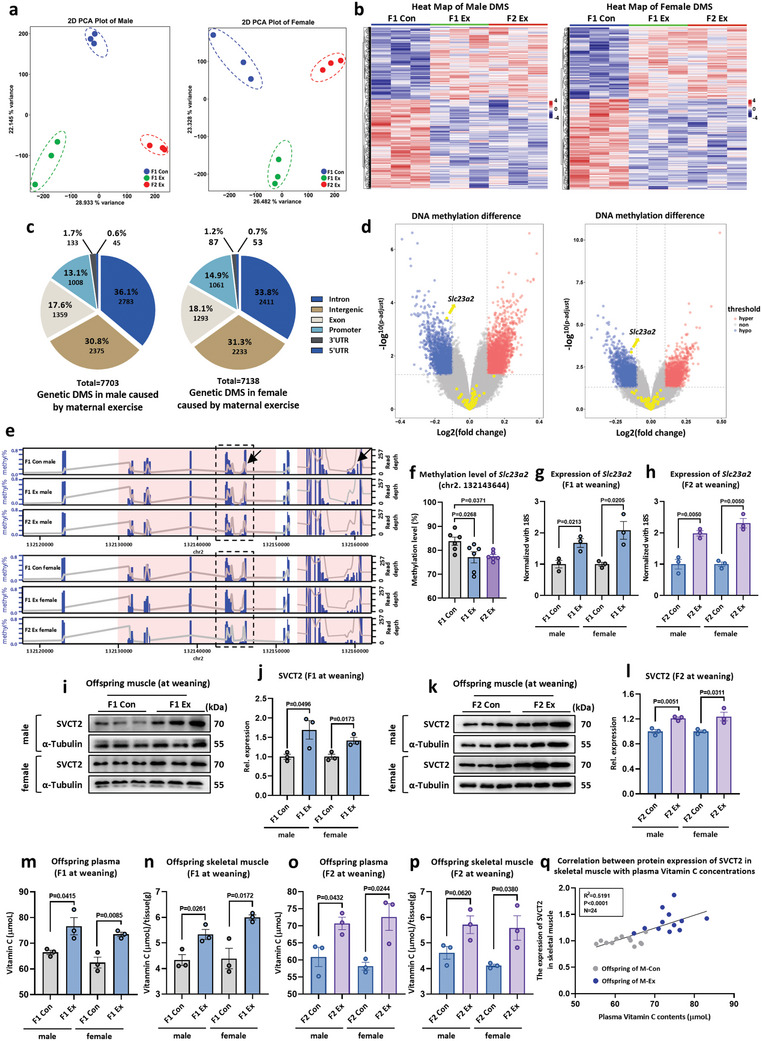
Maternal exercise reprograms DNA methylation of *Slc23a2* and intergenerational inheritance during skeletal muscle development in offspring. a) PCA scores plot indicating discrimination among F1 Con, F1 Ex, and F2 Ex (*n* = 3). b) Clustered heatmap showing the methylation levels across all samples for the hyper and hypo methylated CpG sites (*n* = 3). c) The distribution of common differential methylation sites on different gene elements in the F1 and F2 generations of the M‐Ex compared with the F1 generation of M‐Con, respectively (*n* = 3). d) Volcano plot showing the log2 fold‐difference in methylation level of skeletal muscle (GAS) in male (left) and female (right) mice of F1 generation between M‐Con and M‐Ex groups, through the analysis of MCC‐seq data. Red and blue dots represent upregulated and downregulated DMSs with fold change > 2 and *p* ‐adjusted < 0.05, respectively. Yellow indicates the elements of the *Slc23a2* gene (*n* = 3). e) DNA methylation sequencing results of *Slc23a2*. The blue histogram is the degree of methylation, corresponding to the left ordinate. The gray line is the sequencing depth, corresponding to the right ordinate. The highlighted area on the light red background is the detected DMR, and the abscissa is the chromosome location. f) Methylation levels at the Chr2. 132143644 site in the differential methylation region of *Slc23a2* (*n* = 3). g,h) The expression of *Slc23a2* in skeletal muscle of F1 (g) and F2 (h) generation mice at weaning (*n* = 3). i–l) Western blot analysis of SVCT2 in F1 (i,j) and F2 (k,l) generation skeletal muscle at weaning (*n* = 3). m–p) The concentration of VC in plasma and skeletal muscle at weaning in the F1 (m,n) and F2 (o,p) generation (*n* = 3). q) Pearson correlation coefficient between VC content in the plasma and SVCT2 protein expression in skeletal muscle of F1 and F2 generations in the M‐Con group (gray) and M‐Ex group (blue) (*n* = 24). Statistical analyses were performed using *t*‐test (g–p) and one‐way ANOVA with Bonferroni's post hoc analysis (f). Data are presented as mean ± SEM.

Epigenetic modifications are a fundamental mechanism that exercises and regulates transcriptional changes, referring to phenotypic changes that are not rooted in DNA sequence.^[^ ^35^ ^]^ To investigate the potential reprogramming effects of maternal exercise on offspring DNA methylation, MCC‐Seq was performed on the skeletal muscle of F1 generation mice. Principal component analysis (PCA) (**Figure** **2**a) and the heatmap (Figure 2b) distinctly separated the M‐Con (F1 Con) from the M‐Ex (F1 Ex) groups, implying that maternal exercise can reprogram offspring DNA methylation profiles. In the F1 Ex group, a total of 43641 and 39234 differential methylation sites (DMSs) were identified in males and females, respectively, compared to the F1 Con group (Figure S6a, Supporting Information). Of these DMSs, 5788 in males and 5573 in females were found within CpG islands (CGIs) of the gene promoter region. Further, 3100 genes from males and 2934 genes from females were associated with promoter regions, with 1050 of these genes being shared between both genders (Tables S1 and S2, Supporting Information).

Subsequently, MCC‐Seq was conducted on F2 generation offspring derived from F0 maternal exercise (F2 Ex). The DNA methylation patterns of F2 Ex offspring closely resembled those of F1 Ex mice and were significantly different from those of F1 Con mice (Figure 2a,b). Specifically, the F2 Ex group exhibited 53712 DMSs in males and 39910 DMSs in females compared to the F1 Con group (Figure S6b, Supporting Information). Among these, 7020 DMSs in males and 5790 DMSs in females were located within CGIs of gene promoter regions. In addition, 3410 genes in males and 3125 genes in females were associated with promoter regions, with 1113 genes shared between both genders (Tables S3 and S4, Supporting Information).

To investigate the persistence of CGI methylation changes induced by maternal exercise, the overlap of DMSs between the F1 and F2 generations was examined. A total of 7703 common DMSs were identified in males and 7138 common DMSs in females (Figure 2c). Within these overlapping DMSs, 1008 in males and 1061 in females were located in promoter regions, regulating the expression of 336 and 166 genes, respectively (Tables S5 and S6, Supporting Information). Taken together, these data show that maternal exercise reprograms DNA methylation in offspring skeletal muscle, with a subset of DMSs being stably inherited across generations.

### 2.4 Maternal Exercise Facilitates the Intergenerational Inheritance of DNA Demethylation on the *Slc23a2* Gene in Offspring Skeletal Muscle

The *Slc23a2* gene, which encodes SVCT2 protein,^[^ ^36^ ^]^ was observed to be demethylated in both F1 and F2 generations as a result of F0 maternal exercise, as documented in the DMSs (Figure 2d–f). Correspondingly, both the *Slc23a2* gene (Figure 2g,h) and SVCT2 protein (Figure 2i–l) expression were significantly elevated in F1 and F2 offspring skeletal muscle at weaning. In addition, maternal exercise was observed to enhance the expression of *Gulo* in the liver (Figure S6c,d, Supporting Information) and to increase VC levels in the plasma of F1 and F2 offspring (Figure 2m,o). A robust positive correlation was identified between plasma VC levels and SVCT2 protein in skeletal muscle (Figure 2q). Given that SVCT2 is a high‐affinity VC transporter that regulates tissue accumulation of VC, it was noted that maternal exercise markedly increased the amount of VC in offspring skeletal muscle (Figure 2n,p). Further, maternal exercise‐induced PGC‐1α protein expression in skeletal muscle was found to be positively correlated with VC concentration (Figure S6e, Supporting Information). Taking together, these data demonstrate that maternal exercise upregulates the expression of SVCT2 and enhances VC uptake in offspring's skeletal muscle, suggesting that VC/SVCT2 may be potential mediators of the beneficial effects of maternal exercise on offspring endurance performance.

### 2.5 The Enhanced Endurance Performance Following Maternal Exercise is Dependent on VC

**Figure 3:**
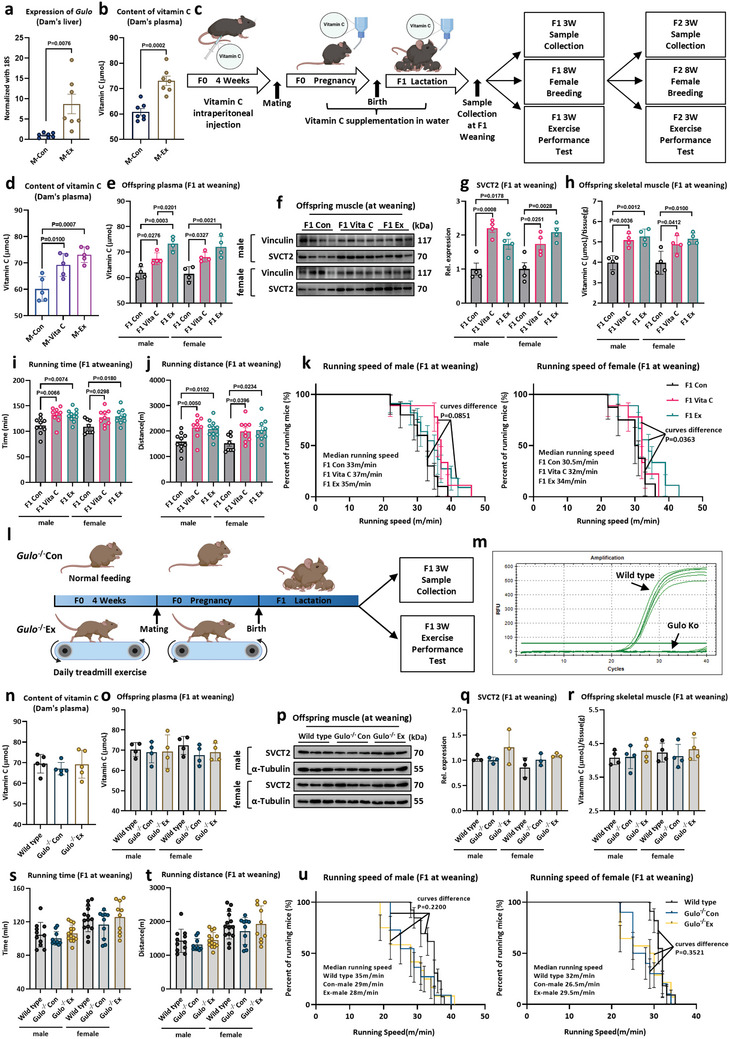
The enhanced endurance performance following maternal exercise is dependent on VC. a,b) Exercise promoted *Gulo* gene expression (a) in the maternal liver and elevated plasma VC content (b) (*n* = 7). c) A schematic diagram of the experiment. Wild‐type female mice were supplemented with VC before and during pregnancy and then obtained F1 generation after mating with wild male mice of the same age. F1 generation mice underwent exercise performance tests at weaning and mated with wild male mice at 8 weeks of age to obtain F2 and F3 generation. d) The content of VC in dam's plasma (*n* = 5). e) The content of plasma VC at weaning in F1 generation (*n* = 4). f,g) Representative images of Western blot analysis of SVCT2 in skeletal muscles of F1 generation (*n* = 4). h) The concentration of VC in skeletal muscle at weaning in the F1 generation (*n* = 4). i–k) The effect of maternal exogenous supplementation of VC before and during pregnancy on the endurance performance of offspring. Running time (i), distance (j), and population plotted against running speed to exhaustion (k) (Male: F1 Con *n* = 10, F1 Vita C *n* = 9, and F1 Ex *n* = 11. Female: F1 Con *n* = 8, F1 Vita C *n* = 9, and F1 Ex *n* = 9). l) A schematic diagram of *Gulo^−/−^* mice maternal exercise. m) The representative diagram of the *Gulo* gene PCR amplification curve of *Gulo^−/−^* mice's liver. n) The content of VC in the Dam's plasma (*n* = 5). o) The content of plasma VC at weaning in F1 generation (*n* = 4). p,q) Representative images of Western blot analysis of SVCT2 in skeletal muscles of F1 generation (*n* = 3). r) The concentration of VC in skeletal muscle at weaning in the F1 generation (*n* = 4). s–u) Effects of maternal exercise in *Gulo^−/−^* mice (the same training protocol as wild‐type mice) on the endurance performance of their offspring at weaning. Running time (s), distance (t), and population plotted against running speed to exhaustion (u) (Male: Wild type *n* = 11, *Gulo^−/−^* Con *n* = 10, and *Gulo^−/−^* Ex *n* = 14. Female: Wild type *n* = 14, *Gulo^−/−^* Con *n* = 10, and *Gulo^−/−^* Ex *n* = 10). Statistical analyses were performed using *t*‐test (a,b), one‐way ANOVA with Bonferroni's post hoc analysis (d,e,g,h,i,j,n,o,q,r,s,t) and the Log‐rank test (k,u). Data are presented as mean ± SEM.

A significant increase in *Gulo* expression was observed in the maternal liver following maternal exercise (**Figure** **3**a), accompanied by elevated plasma VC concentrations in the exercised mothers (Figure 3b). In parallel, an upregulation of *Slc23a2* gene expression was detected in the maternal placenta (Figure S7a, Supporting Information), indicating that maternal exercise may facilitate placental VC translocation, thereby establishing an intrauterine environment rich in VC during fetal development. Given these findings and the heightened VC levels in the plasma and skeletal muscle of the offspring (Figure 2m,n), it was investigated whether VC contributes to the enhancement of endurance performance through exogenous VC supplementation and endogenous VC deficiency (*Gulo* knockout mice) experiments. Specifically, wild‐type female mice received intraperitoneal injections of VC (4 mg g^−1^ body weight) every 2 days for 4 weeks prior to mating and were supplemented with VC through drinking water (18.75 mM) during pregnancy and lactation^[^ ^37^, ^38^ ^]^ (Figure 3c). Maternal exogenous VC supplementation elevated the dam's plasma VC content (Figure 3d) and placental *Slc23a2* gene expression (Figure S7b, Supporting Information), while having no effect on liver *Gulo* gene expression (Figure S7c, Supporting Information). Further investigation showed that exogenous VC administration elevated plasma VC concentration in offspring at weaning and enhanced the expression of SVCT2 in their skeletal muscle (Figure 3e–g), leading to an increase of VC within the skeletal muscle (Figure 3h). Excitingly but not unexpectedly, exogenous VC supplementation considerably enhanced F1 offspring physical fitness, as indicated by prolonged running time to exhaustion, increased total distance, and enhanced maximum running speed (Figure 3i–k), without affecting forelimb grip strength (Figure S7d, Supporting Information). Subsequent analysis revealed that the protein expression of PGC‐1α and genes associated with mitochondrial biogenesis, such as *Ppargc1a*, *Tfam*, *Tfb1* *m*, and *Tfb2* *m*, were highly upregulated in the skeletal muscle of offspring from mothers supplemented with VC (Figure S7e–g, Supporting Information). In addition, immunostaining of skeletal muscle sections for SDH revealed a greater number of positive fibers of offspring (Figure S7h,i  Supporting Information), coupled with an increase in mtDNA copy number (Figure S7j, Supporting Information), resembling the pattern observed in maternal exercise. Strikingly, despite exogenous VC administration being conducted only on F0 mice, the running distance and running time of F2 and F3 females remained significantly higher than those of control mice (Figure S8, Supporting Information). These findings showed that exogenous VC supplementation during pregnancy mimics the beneficial impact of maternal exercise on offspring endurance performance.

In most mammals, VC can be synthesized de novo from glucose in the liver, facilitated by the enzymatic action of *Gulo*. To explore whether the enhancement of endurance performance in offspring following maternal exercise is indeed dependent on VC, the *Gulo^−/−^* mouse model was utilized (Figure 3l). The *Gulo^−/−^* mice were maintained on dietary VC (2 mM) for long‐term survival.^[^ ^37^ ^]^ It was found that exercise did not increase plasma VC level in *Gulo^−/−^* mice due to the absence of *Gulo* enzymatic function (Figure 3m,n). Importantly, although a 4‐week treadmill training remarkably enhanced the endurance performance and maximum running speed of maternal *Gulo^−/−^* mice (9‐week‐old) (Figure S9, Supporting Information), the restriction of VC synthesis resulted in maternal exercise failing to upregulate plasma VC levels in the offspring of *Gulo^−/−^* mice (Figure 3o). Consequently, the SVCT2 expression and VC levels in the offspring's skeletal muscle did not exceed those in control mice (Figure 3p–r). Not surprisingly, the effects of maternal exercise on offspring endurance performance were absent in *Gulo^−/−^* mice, including running time to exhaustion, total distance, and maximum running speed (Figure 3s–u), as well as forelimb grip strength (Figure S10a, Supporting Information). In addition, there was no influence on the mitochondria‐related biomarkers such as PGC‐1α in the skeletal muscle of *Gulo^−/−^* mice offspring at weaning (Figure S10b–d, Supporting Information). Collectively, these data suggested that the improved offspring endurance capacity resulting from maternal exercise was dependent on VC.

### 2.6 Exogenous VC Supplementation May Exert its Effects on Offspring Endurance Performance During Fetal Development

**Figure 4:**
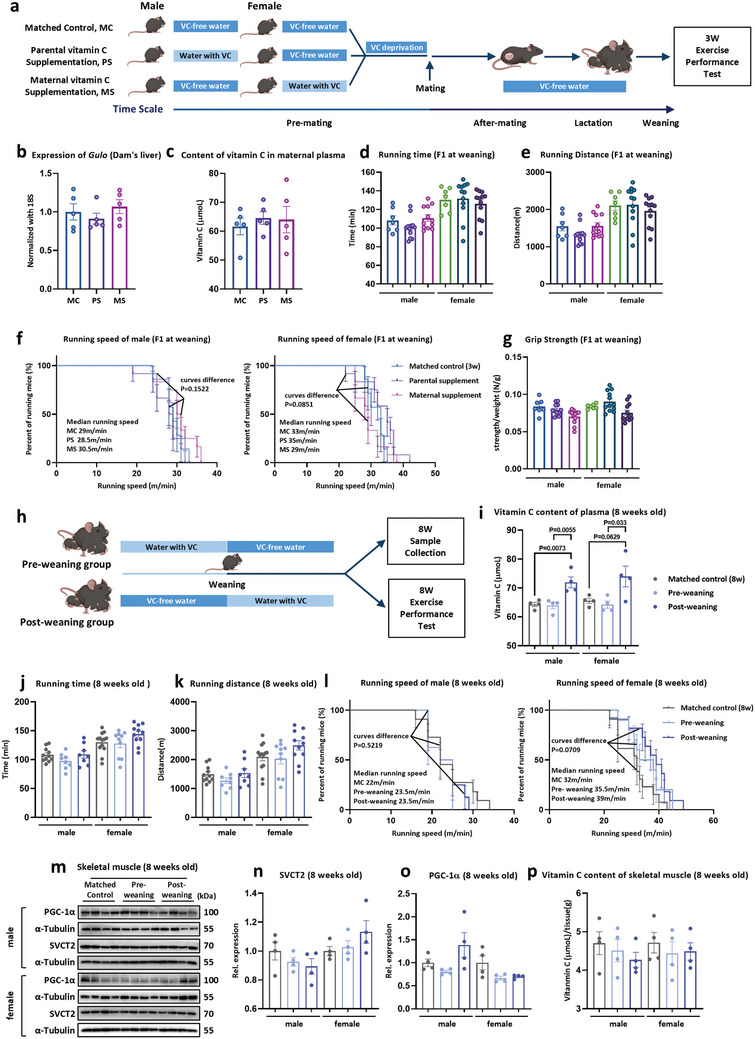
Exogenous VC supplementation during pre‐conception and postnatal development does not affect offspring endurance performance. a) A schematic diagram of the experiment. Maternal (maternal VC supplementation, MS) and paternal (paternal VC supplementation, PS) mice received 4 weeks of exogenous VC supplementation prior to preparation for conception, followed by 2 weeks of VC deprivation. Subsequently, age‐matched wild‐type male mice were mated, and VC withdrawal was maintained during pregnancy and lactation. The exercise performance of the offspring would be measured during the weaning period after birth. b,c) Maternal liver *Gulo* gene expression (b) and plasma VC content (c) (*n* = 5). d–g) The treadmill endurance performance of offspring from parental exogenous VC supplementation during pre‐conception. Running time (d), distance (e), population plotted against running speed to exhaustion (f), and forelimb maximum grip strengths (g) (Male: Matched Control (3W) *n* = 7, PS = 12. MS = 12, Female: Matched Control (3W) *n* = 7, PS *n* = 12, and MS *n* = 12). h) A schematic diagram of the experiment. Postnatal wild‐type mice were supplemented exogenously with VC by drinking water before or after weaning, respectively, and then, their exercise capacity was measured at 8 weeks of age. i) Plasma VC concentrations in mice supplemented with VC before or after weaning (*n* = 4). j–l) Exercise performance testing of exogenous VC supplementation before or after weaning. Running time (j), distance (k), and population plotted against running speed to exhaustion (l) (Male: Matched Control (8W) *n* = 11, pre‐weaning group *n* = 8, and post‐weaning group *n* = 8. Female: Matched Control (8W) *n* = 13, pre‐weaning group *n* = 10, and post‐weaning group *n* = 11). m–o) Representative images of Western blot analysis of SVCT2 (n) and PGC‐1α (o) in skeletal muscle with VC supplementation before or after weaning (*n* = 4). p) The contents of VC in skeletal muscle with VC supplementation before or after weaning (*n* = 4). Statistical analyses were performed using one‐way ANOVA with Bonferroni's post hoc analysis (b–e,g,i–k,n–p) and the Log‐rank test (f,l). Data are presented as mean ± SEM.

The exogenous VC supplementation experiments, shown in Figure 3, covered three periods: pre‐conception, gestation, and lactation. This comprehensive approach made it challenging to pinpoint the specific developmental stage during which VC influences offspring endurance performance, whether during gametogenesis, embryogenesis, or postnatal muscle growth. First, to evaluate the impact of exogenous VC supplementation on gametogenesis, VC was provided exclusively to the parents during the pre‐conception period. Maternal VC supplementation (MS) and paternal VC supplementation (PS) groups received VC supplementation (18.75 mm in drinking water) for 4 weeks prior to conception preparation. This was followed by 2 weeks of VC deprivation, after which the VC concentration was restored to normal (Figure S11a, Supporting Information). Subsequently, these mice were mated with age‐matched wild‐type mice and maintained VC withdrawal during pregnancy and lactation (**Figure** **4**a). It was observed that the mRNA expression of *Gulo* in the liver and the plasma VC content of dams did not change with VC supplementation during pre‐conception period (Figure 4b,c). In addition, neither maternal nor paternal pre‐pregnancy VC supplementation affected the endurance performance of offspring, as evidenced by unchanged running time to exhaustion, total distance, maximum running speed, and forelimb grip strength (Figure 4d–g). These findings show that VC did not enhance the endurance performance of offspring through gametogenesis.

Next, VC supplementation was administered during lactation (pre‐weaning), post‐weaning (from weaning to 8 weeks of age), and adulthood (from 9 to 12 weeks of age), with age‐matched wild type mice serving as controls (8 weeks of age or 12 weeks of age) (Figure 4h; Figure S12a, Supporting Information). Both pre‐ and post‐weaning VC supplementation failed to influence physical fitness, as demonstrated by unchanged running time to exhaustion, total distance, and maximal running speed (Figure 4j–l). Similarly, no significant effects on physical fitness were observed when VC was administered during adulthood (Figure S12c–e, Supporting Information). Despite increases in plasma VC content following both post‐weaning and adult VC supplementation (Figure 4i; Figure S12b, Supporting Information), the protein expression of SVCT2 and PGC‐1α, as well as the VC content in skeletal muscle, showed no significant alterations with either supplementation regimen (Figure 4m–p; Figure S12f–i, Supporting Information). These data showed that VC does not enhance endurance performance in offspring after birth. Collectively, the data presented so far suggest that the beneficial effects of VC on offspring endurance performance are exerted during fetal development rather than during gametogenesis or postnatal growth.

### 2.7 VC Treatment During Early Myogenesis Ex Vivo Upregulates Mitochondrial Biogenesis and Oxidative Fiber Differentiation

**Figure 5:**
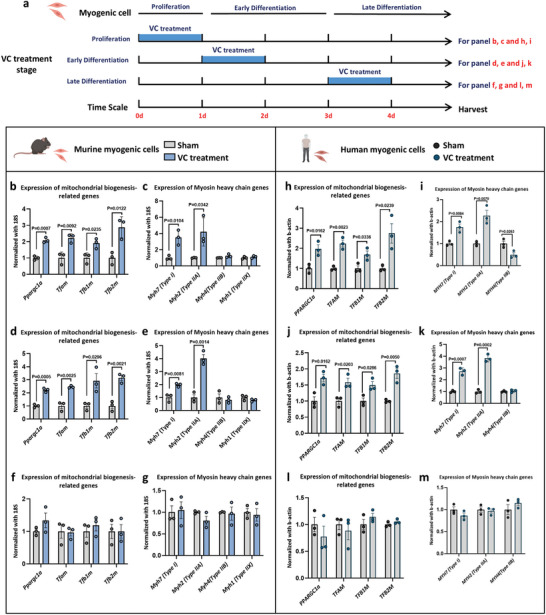
Exogenous VC treatment upregulates mitochondrial biogenesis and differentiation propensity of oxidized fibers in myogenic cells. a) Schematic showing exogenous VC supplementation during myogenic cell proliferation, early and late differentiation for 24 h, and then, cell samples were collected after 4 days of differentiation. b–g) Gene expression related to mitochondrial biogenesis (b,d,f) and myosin isoform (c,e,g) was detected in murine myogenic cells supplemented with VC during proliferation (b,c), early differentiation (d,e), and late differentiation (f,g) (*n* = 3). h–m) Genes expression related to mitochondrial biogenesis (h,j,l) and myosin isoform (i,k,m) were detected in human myogenic cells supplemented with VC in proliferation (h,i), early differentiation (j,k), and late differentiation (l,m) (*n* = 3). Statistical analyses were performed using *t*‐test (b–m). Data presented as means ± SEM.

To confirm the specific role of VC on skeletal muscle development during fetal stages, a primary myogenic cell *ex vivo* differentiation model was employed to simulate the embryonic myogenesis process and examine the effect of VC on mitochondrial biogenesis and the tendency of *MyHC* genes expression. Myogenic cells were treated with VC (300 µM) during distinct phases: proliferation, early differentiation, and late differentiation, to identify the critical window for VC‐induced alternations in myofiber composition and mitochondrial biogenesis (**Figure** **5**a). The results demonstrated that VC supplementation during proliferation and early differentiation phases increased the expression of mitochondria biogenesis‐related genes, including *Ppargc1a*, *Tfam*, *Tfb1* *m*, and *Tfb2* *m* (Figure 5b,d). In contrast, VC treatment during the late differentiation stage did not affect the expression of these genes (Figure 5f). Further, VC supplementation during proliferation or early differentiation promoted the differentiation trend toward oxidative fibers, as evidenced by the upregulation of *Myh7* and *Myh2* expressions (Figure 5c,e). No significant changes in *MyHC* isoform expression were observed with VC supplementation at the late differentiation stage compared with the non‐supplemented group (Figure 5g). To determine whether the effects of VC on myogenic cell differentiation are conserved in humans, similar in *ex vivo* experiments were conducted using human myogenic cells. The findings mirrored those observed in murine myogenic cells, with VC treatment during early myogenesis enhancing mitochondrial biogenesis and promoting oxidative fiber differentiation (Figure 5h–m).

Further, to explore whether observed effects of VC are contributable to its anti‐oxidative properties, other antioxidants were evaluated to determine if they could enhance mitochondrial biogenesis and oxidative fiber differentiation during the proliferation stage of myogenic cells in vitro. Three specific antioxidants were evaluated: *Ferulic acid* (50 µm), *Quercetin* (50 µM), and *Cyanidin* (10 µM) (Figure S13a, Supporting Information).^[^ ^39^, ^40^, ^41^ ^]^ Although these antioxidants, particularly *Cyanidin*, were capable of facilitating mitochondrial biogenesis in myogenic cells during proliferation (Figure S13b, Supporting Information), they did not upregulate mitochondrial biogenesis in myotubes or promote oxidative fiber differentiation to the same extent as VC (Figure S13c,d, Supporting Information). Collectively, these data indicate that VC treatment during the early stages of myogenesis is pivotal for elevating mitochondrial biogenesis and promoting the differentiation of oxidative fibers in myotubes, effects that are independent of VC's antioxidative properties. This underscores the critical window during fetal development when VC exerts its beneficial effects on skeletal muscle physiology, contributing to enhanced endurance performance observed in offspring.

### 2.8 Maternal Exercise May Enhance Offspring Endurance Performance Through VC‐TET2‐SVCT2 Pathway

**Figure 6:**
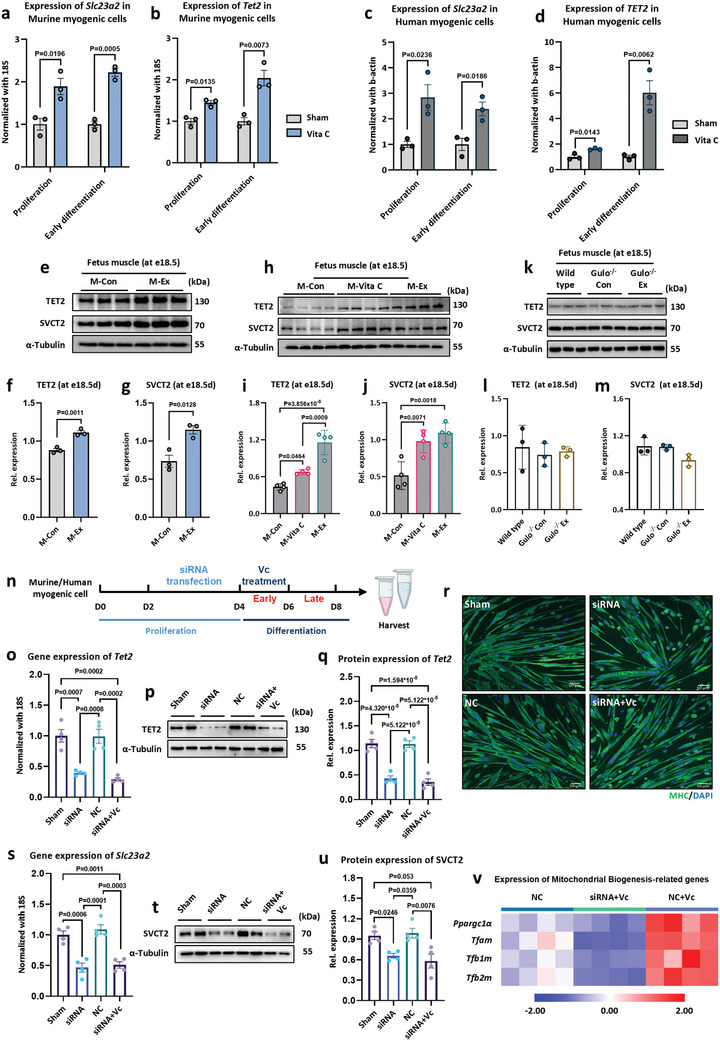
Maternal exercise and exogenous VC supplementation enhance the endurance performance of offspring by upregulating the expression of TET2 during fetal development. a–d) *Slc23a2* and *Tet2* gene expression in murine (a,b) and human (c,d) myogenic cells supplemented with VC during proliferation or early differentiation stage (*n* = 3). e–m) Representative images of Western blot analysis of TET2 (f,i,l) and SVCT2 (g,j,m) in the fetal skeletal muscle of M‐Con and M‐Ex dams (e–g) (*n* = 3), M‐Con, M‐vita C, and M‐Ex dams (h–j) (*n* = 4), *Gluo* ^‐/‐^ Con, and *Gluo* ^‐/‐^ Ex mice (k–m) (*n* = 3). n) A schematic diagram of siRNA transfection experiment. The experiment consists of four groups: the Sham group with normal culture (Sham), the NC group with Negative control (NC), the siRNA group with *Tet2* knockdown using siRNA during the proliferation phase followed by normal differentiation (siRNA), and the siRNA+VC group with *Tet2* knockdown using siRNA during the proliferation phase followed by VC treatment during the early differentiation phase (siRNA+VC). o–q) The gene expression of *Tet2* (o) and representative images of Western blot analysis of TET2 (p,q) after siRNA transfection of myogenic cells (*n* = 4). r) Representative images of the effect after siRNA transfection on differentiation of myogenic cells; the scale bar represents 50 µm. s–u) The gene expression of *Slc23a2* (s) and representative images of Western blot analysis of SVCT2 (t,u) after siRNA transfection of myogenic cells (*n* = 4). v) The gene expression related to mitochondrial biogenesis after siRNA transfection of myogenic cells (*n* = 4). Statistical analyses were performed using *t*‐test (a–d and f–g) and one‐way ANOVA with Bonferroni's post hoc analysis (i,j,l,m,o,q,s,u,v). Data are presented as mean ± SEM.

It was observed that maternal exercise and exogenous VC supplementation significantly upregulated the expression of *Slc23a2* gene and SVCT2 protein in the skeletal muscles of offspring (Figures 2g,j and 3f,g). However, in *Gulo^−/−^* mice, maternal exercise did not elevate the SVCT2 level (Figure 3p,q), suggesting that the upregulation of SVCT2 in the offspring of exercised mice is VC‐dependent. Meanwhile, in the *ex vivo* model, VC treatment upregulated the expression of *Slc23a2* in both murine and human myogenic cells during the proliferation and early differentiation stages (**Figure** **6**a,c), whereas no significant impact was observed when VC was applied during the later stage of differentiation (Figure S14a,g, Supporting Information). This indicates that the enhancement of mitochondrial biogenesis in myogenic cells and the differentiation toward oxidative fibers by VC occurs during the early development stage of fetal skeletal muscle. Moreover, it was verified that the expression of *Slc23a1* remained unchanged at different stages of in vitro myogenic cell culture following VC treatment (Figure S14b,h, Supporting Information). These results suggest that the improvement in skeletal muscle endurance performance of offspring mice induced by maternal exercise or exogenous VC supplementation is likely related to the reprogramming of the *Slc23a2* gene by VC.

Given that maternal exercise affects skeletal muscle DNA methylation patterns (Figure 2a–f), specifically *Slc23a2* gene in this study (Figure 2f), the expression levels of *Tets* and *Dnmts*, the essential enzymes for DNA methylation regulation,^[^ ^42^ ^]^ were measured. It was found that the mRNA expression level of *Dnmts*, *Tet1*, and *Tet3* was not changed in myogenic cells with VC treatment (Figure S14c–e,i–k, Supporting Information). However, VC treatment during proliferation and early differentiation stages highly upregulated the gene expression of *Tet2* in both murine and human myogenic cells (Figure 6b,d), whereas VC treatment during the late differentiation stage had no effect on TET2 expression (Figure S14f,l, Supporting Information). In addition, it was observed that other antioxidants, such as *Quercetin*, *Cyanidin*, and *Ferulic acid*, did not impact *Tet2* expression when applied during the proliferation period (Figure S13e, Supporting Information).

Subsequently, the protein expression of TET2 in fetal skeletal muscle at embryonic day 18.5 (e18.5) was examined. In alignment with the results observed in myogenic cells, significantly elevated protein levels of TET2 and SVCT2 were detected in fetal skeletal muscle of the M‐Ex group compared to the M‐Con group (Figure 6e–g). Moreover, the catalytic activity of TET dioxygenases for 5mC oxidation requires Fe^2+^ and α‐KG as cofactors.^[^ ^43^ ^]^ A‐KG is a crucial intermediate metabolite produced by isocitrate dehydrogenases (*Idh1/2*).^[^ ^44^ ^]^ Data revealed that maternal exercise increased the expression of *Idh1* and *Idh2* in fetal skeletal muscle (Figure S15a, Supporting Information). Similarly, following maternal VC supplementation, elevated expression of TET2, SVCT2 (Figure 6h–j) and *Idh1/2* (Figure S15b, Supporting Information) were observed in e18.5 fetal muscle. In contrast, maternal exercise in *Gulo^−/−^* mice did not significantly affect the expression of TET2 and SVCT2 in fetal skeletal muscle at e18.5 (Figure 6k–m). Further, the protein expression of TET2 and SVCT2 did not change in fetal skeletal muscle at e18.5 with parental VC supplementation during pre‐conception (Figure S15c–e, Supporting Information). Taken together, these data indicate that exercise promotes the synthesis and secretion of VC by the maternal liver and promotes the expression of TET2 and SVCT2 during fetal skeletal muscle development.

To further dissect the interaction among VC, TET2, and SVCT2, *Tet2* was silenced in myogenic cells using siRNA, followed by treatment with exogenous VC during differentiation to assess the impact of VC treatment on *Slc23a2* expression and mitochondrial biogenesis in TET2‐deficient myogenic cells (Figure 6n). Compared to the Sham and Negative Control (NC) group, siRNA (20 nM) treatment significantly attenuated both the gene and protein expression of TET2 in myogenic cells (Figure 6o–q), concomitant with a marked decrease in expression of *Slc23a2* and SVCT2 (Figure 6s–u), while not affecting myogenic cell differentiation (Figure 6r). Moreover, the loss of TET2 expression rendered VC treatment ineffective in upregulating the protein expression of SVCT2 in myotubes (Figure 6s–u), as well as the expression of mitochondrial biogenesis‐related genes in murine myogenic cells (Figure 6v). Consistently, the effects of VC on the expression of *Slc23a2* and mitochondrial biogenesis‐related genes were blocked by TET2 silencing in human myogenic cells (Figure S15f–j). Collectively, these results indicate that VC upregulates SVCT2 expression via TET2 during skeletal muscle development, thereby enhancing VC uptake in myogenic cells, promoting mitochondrial biogenesis, and ultimately, improving endurance performance in the offspring.

## 3 Discussion

**Figure 7:**
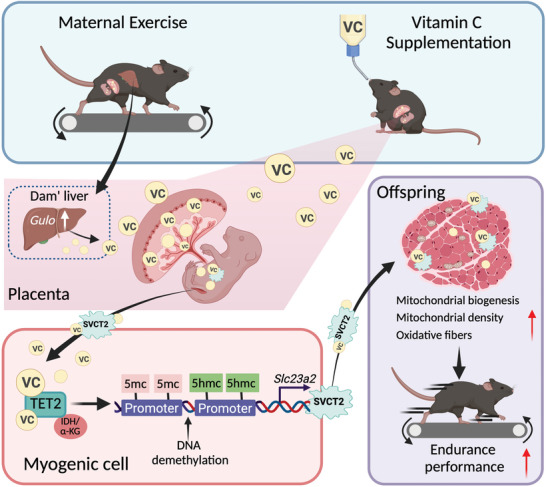
Working model. Proposed regulatory model for intergenerational improvement of endurance performance in offspring by maternal exercise or exogenous VC supplementation in mice. Image created with BioRender. com, with permission.

In this study, we identified that maternal exercise enhances offspring endurance performance through a VC‐dependent mechanism. We found that maternal exercise upregulates the expression of *Gulo* in the maternal liver, promoting VC synthesis and secretion. VC is subsequently transported through the placenta into the fetus, where it promotes the expression of TET2, which induces targeted DNA demethylation and gene expression during fetal skeletal muscle development. This results in elevated mitochondrial biogenesis and differentiation tendency of oxidative fibers in offspring skeletal muscle (**Figure** **7**). Interestingly, we observed that these benefits extend to the F2 and F3 generations, indicating stable, inter‐, and trans‐generational effects. Further, exogenous VC supplementation during pregnancy is found to mimic the beneficial effects of maternal exercise, significantly improving offspring endurance performance and skeletal muscle mitochondrial biogenesis. These findings suggest a potential health‐promoting strategy wherein sufficient maternal physical activity during pregnancy effectively improves skeletal muscle health and physical fitness in offspring.

VC is a water‐soluble antioxidant commonly used as an oral supplement to support the endogenous antioxidant defense system. It is widely believed to reduce oxidative stress, minimize muscle damage, and improve exercise performance.^[^ ^45^ ^]^ However, the effects of VC supplementation on physical performance remain inconsistent and context‐dependent.^[^ ^46^ ^]^ VC deficiency in skeletal muscles is known to cause muscle atrophy, accompanied by the upregulation of muscle atrophy‐related genes and decreased physical performance.^[^ ^47^ ^]^ In non‐exercising individuals, VC supplementation did not affect resting skeletal muscle antioxidant enzyme levels.^[^ ^48^ ^]^ In addition, daily oral consumption of 1 g of VC over a 4‐week high‐intensity interval training period did not hinder training‐induced improvements in exercise performance among recreationally active males.^[^ ^49^ ^]^ In contrast, long‐term supplementation with combined VC and vitamin E (VE) had been shown to negate the health‐promoting effects of physical exercise and oxidative stress adaptation in humans.^[^ ^50^ ^]^ Further, acute supplementation with VC and VE failed to attenuate skeletal muscle oxidative stress or enhance the expression of mitochondrial biogenesis markers.^[^ ^51^ ^]^ These findings suggest that the role of VC supplementation in skeletal muscle adaptations to exercise remains controversial, particularly when combined with other antioxidants. Studies examining the effects of VC supplementation alone on skeletal muscle adaptations to exercise training are still limited.^[^ ^52^ ^]^ In the present study, we observed that exogenous VC supplementation at different developmental stages postnatally had no effect on exercise performance. In contrast, maternal exogenous VC supplementation during pregnancy significantly improved offspring endurance performance, underscoring the critical prenatal period for VC to influence performance outcomes.

VC exists in two forms: its reduced state and its oxidized form. All known biological functions of VC are associated with its reduced state, which is absorbed from the intestine via the sodium‐dependent vitamin C transporter 1 (SVCT1). Tissue distribution of VC from the bloodstream is regulated by tissue‐specific SVCT2.^[^ ^28^, ^53^ ^]^ SVCT2 has a higher affinity for VC than SVCT1, being approximately half‐saturated at the low end of the normal range of plasma ascorbate concentrations. Upregulated SVCT2 increases the maximal rate of ascorbate uptake in cells.^[^ ^28^ ^]^ In this study, we identified demethylation of *Slc23a2* gene in offspring's skeletal muscle followed by maternal exercise (Figure 2e,f), leading to the upregulation of SVCT2 expression and enhanced VC uptake in offspring skeletal muscle (Figure 2i–l). Notably, this upregulation of SVCT2 was observed exclusively during fetus skeletal muscle development (Figure 6e,h). Consequently, the intracellular concentration of VC was determined by the amount of SVCT2. Supplementation of VC at other developmental stages, although it increased plasma VC levels (Figure 4i; Figure S12b, Supporting Information), did not result in an increase in SVCT2 expression and VC content in skeletal muscle (Figure 4m,n; Figure S12f,g, Supporting Information), indicating that SVCT2 expression does not correspond directly to plasma VC concentration during postnatal development or in adulthood (Figure 4j–l; Figure S12c–e, Supporting Information). In addition, plasma VC concentration returned to normal levels 48 h after VC withdrawal (Figure S11a, Supporting Information), implying that maternal exogenous VC supplementation should be maintained throughout the entire pregnancy period to achieve sustained benefits.

VC also serves as a co‐factor for many α‐KGDDs, including histone demethylases and the TET‐class of cytosine hydroxylases, making VC an epigenetic compound with broad significance in various pathophysiological contexts.^[^ ^22^, ^24^, ^54^ ^]^ Clinic trials have shown that VC prevents DNA methylation changes in the offspring of pregnant smokers and improves offspring lung function.^[^ ^55^, ^56^ ^]^ In our study, we speculated that certain α‐KGDDs could be regulated after VC treatment. Thus, we examined the gene expression of *Tets* and *Dnmts*, which are essential enzyme families that regulate DNA methylation during fetal development in myogenic cells treated with VC at different differential stages. We identified that VC upregulated TET2 gene expression in murine and human myogenic cells during proliferation and early differentiation stages.

Next, we silenced TET2 in myogenic cells using siRNA and observed that loss of TET2 function inhibited exogenous VC treatment from upregulating mitochondrial biogenesis‐related gene expression (Figure 6v; Figure S15j, Supporting Information). In addition, siRNA inhibition of TET2 expression directly led to decreased SVCT2 gene and protein expression in both murine and human myogenic cells, with exogenous VC treatment unable to restore SVCT2 levels (Figure 6s–u; Figure S15g,i, Supporting Information), these data suggest that VC‐promoted myogenic mitochondrial biogenesis depends on TET2‐activated SVCT2 expression. This finding is consistent with our *ex vivo* results, where the maternal exercise or VC supplementation during pregnancy upregulated TET2 and SVCT2 expression in fetal skeletal muscle during intrauterine development, enhancing offspring endurance performance. Conversely, providing exogenous VC to the parent during the pre‐conception period or maternal exercise in the *Gulo^−/−^* mice had no effect on TET2 and SVCT2 expression in fetal skeletal muscle and offspring endurance performance. Together, these findings reveal the pivotal role of the TET2‐SVCT2 signaling in maternal exercise in improving offspring endurance performance and identify that SVCT2 expression in skeletal muscle cell is TET2‐dependent.

AMPK is a heterotrimeric complex with central roles in cellular energy sensing, metabolism, and exercise adaptations.^[^ ^57^ ^]^ Recent studies have shown that maternal exercise activates AMPK and increases α‐KG,^[^ ^14^, ^15^ ^]^ a rate‐limiting cofactor required for TET‐catalyzed DNA demethylation, thereby promoting gene demethylation in F1 offspring. Concurrently, VC enhances TET enzyme activity by maintaining Fe^2+^ in its reduced state, an essential condition for TET‐mediated reactions. Consequently, VC and α‐KG are both critical to TET‐mediated DNA demethylation, particularly in the initial oxidation step where TET enzymes convert 5mC to 5hmC. Moreover, maternal exercise has been shown to regulate liver glycolipid metabolism homeostasis and skeletal muscle function in offspring via an AMPK – α‐KG pathway.^[^ ^14^, ^15^ ^]^ In our study, we found that maternal exercise or maternal VC supplementation during pregnancy can shape epigenetic reprogramming in fetal skeletal muscle development, highlighting VC as a key factor in the intergenerational transmission of exercise benefits. However, the precise mechanism by which VC and AMPK interact to modulate epigenetic changes remains unclear, and whether AMPK activation combined with VC supplementation can more effectively simulate maternal exercise benefits warrants further investigation.

While many studies have confirmed the impact of maternal exercise on the health of first‐generation offspring,^[^ ^13^, ^17^, ^58^, ^59^ ^]^ we demonstrate that maternal exercise has intergenerational genetic effects to improve the endurance performance of second and third generation male and female offspring. To the best of our knowledge, this is the first study to report the trans‐generational influence of maternal exercise, underscoring the significance of promoting physical activity during pregnancy for long‐term health benefits across multiple generations. However, despite the presence of some instances of heritable phenotypic variation,^[^ ^60^, ^61^ ^]^ transgenerational epigenetic inheritance in mammals remains a debated subject. Recently, a study demonstrated that DNA methylation of promoter associated CpG islands (CGIs) can be transmitted from parents to their offspring in mice,^[^ ^62^ ^]^ providing evidence supporting transgenerational epigenetic inheritance in mammals.

In conclusion, VC is an essential micronutrient for human health and acts as a cofactor for TET2 in regulating fetal skeletal muscle mitochondrial biogenesis and the differentiation of oxidative fibers, thereby improving the endurance performance of offspring. Moreover, maternal exercise‐induced heritable DNA demethylation of the *Slc23a2* gene may be a potential mechanism for transgenerational epigenetic inheritance related to endurance performance. Together, this study has uncovered the molecular and epigenetic mechanisms underlying the profound effects of maternal exercise on offspring physical fitness, providing insight into the development of intervention strategies for maternal and child health.

While this study preliminarily elucidated that VC and its transporters are the potential mechanisms mediating the intergenerational inheritance of endurance performance, several limitations warrant consideration. First, we investigated exercise effects on very young female mice (prepuberal) and did not investigate the potential influence of post‐puberal maternal exercise and paternal exercise on offspring fitness. Exploring the effects of prepuberal or post‐puberal parental exercise could provide a more comprehensive understanding of the intergenerational effects of physical activity. Second, we did not assess whether the transgenerational genetic effects of increased endurance performance in F2 generation could be linked to the altered DNA methylation in germ cells of F1 mice. Third, the detailed mechanism by which VC induces TET2 recruitment to fetal skeletal muscle *Slc23a2* and mitochondrial biogenesis related genes for DNA demethylation remains to be further studied. Fourth, the optimal dosage and timing of VC supplementation were not examined in this study. Further research is needed to determine the most effective therapeutic interventions based on VC supplementation. Last, our study focused specifically on the effects of aerobic exercise on offspring exercise capacity. The impact of other forms of exercise on offspring fitness remains an important area for future investigation.

## 4 Experimental Section

### General Animal Information

All experimental procedures and animal care were approved by the Institutional Animal Care and Use Committee (IACUC) of the South China Normal University (Permit number SCNU‐SPT‐2022‐015). C57BL/6J mice and Kunming mice were purchased from the Guangdong Provincial Medical Laboratory Animal Center (Stock number SCXK2016‐0041). *Gulo* knockout mice were obtained from the Shanghai Model Organisms Center (Stock number N7‐4556). All mice were housed in a specific barrier‐protected humidity‐controlled facility under a 12‐h light–dark cycle at 23 °C (±1 °C) and were provided with food and water ad libitum.

### Exercise Training

Four‐week‐old female C57BL/6J mice were randomly allocated to either the sedentary (M‐Con) or exercise (M‐Ex) group. Mice in the M‐Ex group underwent daily exercise following the principles of progressive loading in intensity and duration. During the adaptation period, mice were subjected to a flat degree of treadmill exercise at 10 m min^−1^ for 20 min, three times/week to acclimatize to the treadmill environment. Then, mice were subjected to treadmill training for 50 min daily, 6 days per week, over a four‐week period. The treadmill running commenced at a speed of 18 m min^−1^, increasing by 2 m min^−1^ weekly, and reaching up to 24 m min^−1^ by the end of the training period. The exercise regimen was administered as follows: warming up at 5 m min^−1^ for 10 min, main exercise at 18–24 m min^−1^ for 40 min, and cooling down at 5 m min^−1^ for 10 min. After the 4‐week training period, exercised or sedentary female mice (9 weeks old) were sequentially paired with age‐matched wild‐type male mice (9 weeks old) to ensure consistent paternal influence across groups. To minimize potential paternal effects on offspring, bedding materials containing the scent of male mice were introduced to the cages of female mice in both the M‐Con and M‐Ex groups 1 day prior to mating. Initially, the wild‐type males were placed in the M‐Con female cages, and after mating was confirmed by the presence of a vaginal plug (designated as embryonic day 0.5, E0.5), males were transferred to the M‐Ex female cages. This procedure maintained controlled experimental conditions while reducing paternal confounding variables. The exercise protocol during pregnancy entailed a daily decrease in running speed by 1 m min^−1^ from 24 m min^−1^, six times per week, for 50 min per session. Exercise training was terminated on E18.5.^[^ ^13^, ^14^ ^]^ All training sessions were conducted in the afternoon (between 16:00 and 17:00). M‐Con group mice were placed in the same treadmill environment. However, the treadmill was not operational, thereby excluding the influence of diet, water intake, and environmental factors caused by daily training on the experimental results. The exercise regimen of *Gulo* Knockout mice and Kunming mice was identical to that of the C57BL/6J mice. For the F2 and F3 generation mice, female mice within the F1 Con group and the F1 Ex group were raised under normal conditions until they reached 8 weeks of age. Subsequently, they were paired with age‐matched wild‐type male mice, and mating was verified by the presence of a vaginal smear. Normal feeding was sustained during pregnancy until the F2 generation mice were acquired. The method of obtaining the F3 generation mice was identical to that of the F2 generation mice.

### Endurance Exercise Capacity Test

The treadmill exercise was initiated at 10 m min^−1^ for 40 min and was increased by 1 m min^−1^ every 10 min until it reached 13 m min^−1^. Subsequently, the speed was increased by 1 m min^−1^ every 5 min until exhaustion. Exhaustion was defined as when a mouse remained on the treadmill’ s shock grid for over 30 s or displayed insensitivity to external stimuli. Running time and running distance were recorded.^[^ ^63^, ^64^ ^]^ The endurance exercise capacity test, maximal running speed test, and grip strength test were completed within 1 week.

### Maximal Running Speed Test

The exercise began with a 5‐min warm‐up at a speed of 10 m min^−1^. Thereafter, the running speed increased by 3 m min^−1^ every 3 min until it reached 28 m min^−1^. Thereafter, the speed was further increased by 1 m min^−1^ each minute until the mice were unable to continue.^[^ ^15^ ^]^

### Maximal Grip Strength Test

The grip strength test was performed to measure the forelimb grip strength with a digital grip‐strength meter. The grip strength test was repeated five times, and the maximal value was selected as the forelimb grip strength, then, normalized by body weight.^[^ ^65^ ^]^

### Voluntary Exercise Study

At the weaning of offspring, male and female animals were individually housed in cages, each equipped with a running wheel (11 cm diameter) and an odometer for 1 week, measuring the daily running distance to indicate the physical activity tendency of the mice.

### Open Field Test

On the day of the open field testing, mice were transported to the behavioral testing room and allowed to habituate for at least 30 min before testing. Mice were placed in the center of the open field (50 cm × 50 cm white acrylic board with an average of 25 independent square areas) and allowed to explore the open field for 5 min. ANY‐maze software (Stoelting Company, USA) was applied to track the animals and analyze the data. The total distance travel and speed were recorded and analyzed.^[^ ^66^ ^]^

### VC Administration

VC administration was carried out using L‐Ascorbic acid (Sigma–Aldrich, A92902) or 0.9% NaCl as the control. Prior to mating, female mice in the VC‐supplemented group received intraperitoneal injections of VC every 2 days at a dosage of 4 mg g^−1^ body weight. Following mating, VC supplementation (18.75 mM) was provided via drinking water until birth. The drinking water containing the VC was replaced every other day with freshly prepared solutions to ensure consistent dosing. In addition, the daily water intake of C57BL/6J mice was monitored over a 7‐day period, and it was determined that VC supplementation did not affect water consumption (Figure S11b, Supporting Information). For *Gulo^−/−^* mice, VC was administered through drinking water at a concentration of 2 mm to maintain physiological activity.^[^ ^22^ ^]^ This supplementation was continued to ensure that *Gulo^−/−^* mice received adequate VC, compensating for their inability to synthesize VC endogenously.

### Genotyping of Gulo‐KO Mice

Genotyping was carried out as follows: In brief, a portion of the mouse tail tips was removed using a sterile scalpel and incubated with a mixture of 500 µL of lysate and 50 µL of proteinase K solution at 56 °C in a hybridization furnace overnight. Upon reaching room temperature, samples were centrifuged for 10 min at 12 000 rpm, and the supernatant was collected. To this, 1 mL of absolute ethanol was added and gently shaken until flocculent precipitation was observed. Following centrifugation, the samples were left to dry at room temperature for 15 min to collect genomic DNA.

PCR amplification was executed using primers for the Gulo WT allele (forward, 5′‐ AATGGAAAGGGATGCCTGGG‐3′; mutant reverse, 5′‐TTCCCGCTCCACCAAGGTGAAG‐3′; and wild‐type reverse, 5′‐GCTACAGCAGGAGATGGGGAGATT‐3′). The PCR reaction was performed with Vazyme Taq Plus master mix (Vazyme, P212), 3 min at 94 °C, followed by cycles of 30 s at 94 °C, 30 s at 58 °C, and 30 s at 72 °C, culminating with a 5‐min phase at 72 °C, and eventually held at 12 °C. Both WT and KO primer PCRs consisted of 35 cycles.

PCR products were run on a 100 bp Plus DNA Ladder (TransGen Biotech, BM311‐01). Expected PCR products for WT alleles were 655 base pairs, whereas KO alleles were anticipated to yield PCR products of 266 base pairs in size.

### Quantitative RT‐PCR Analysis

Total RNA was extracted from the offspring liver, skeletal muscle, and the placenta of E18.5 from Dams by using RNAiso Plus reagent (Takara, 9109). Total RNA (1 µg) was reverse transcribed with a PrimeScript TM RT reagent Kit with gDNA Eraser (Takara, RR047A). For human and murine myogenic cells, 1–5 × 10^5^ cells were lysed in 1 mL RNAiso Plus reagent for 5 min at room temperature, then treated in the same way as tissue samples. Quantitative RT‐PCR reactions were conducted using TB‐Green Premix Ex Taq (Takara, RR420A). 18s rRNA was used for normalization, and relative mRNA was calculated by a comparative method (2^−ΔΔCt^). Primer sequences are listed in Table S7, Supporting Information.

### Mitochondrial DNA Content

The copy number of mitochondrial DNA (mtDNA) was quantified by measuring NADH dehydrogenase subunit 1(*Nd1*: forward, 5′‐ CACTATTCGGAGCTTTACG‐3′; reverse, 5′‐ TGTTTCTGCTAGGGTTGA‐3′) and normalized to the nuclear DNA lipoprotein lipase (*Lpl*: forward, 5′‐ GAAAGGGCTCTGCCTGAGTT‐3′; reverse, 5′‐ TAGGGCATCTGAGAGCGAGT‐3′) gene (genomic DNA) using RT‐PCR.^[^ ^15^ ^]^

### Transmission Electron Microscopy (TEM)

Mice were euthanized and perfused with sodium phosphate buffer (PB, 100 mM, pH 7.4), followed by a pre‐fixation solution consisting of 2.5% v/v glutaraldehyde and 1% paraformaldehyde in PB. The tibialis anterior (TA) muscle was dissected, sectioned into small pieces, and fixed in the same pre‐fixation solution overnight at 4 °C. After rinsing with PB, the tissue samples were immersed in 0.2 M imidazole in PB for 15 min, followed by post‐fixation with 1% osmium tetroxide in PB. Following another rinse with high‐purity water, the samples were stained with 1% aqueous lead acetate at 4 °C overnight. Gradient dehydration was performed by sequentially increasing the concentration of acetone, after which the samples were embedded in epoxy resin and polymerized at 60 °C for 24 h. Ultrathin sections were prepared using a Leica UC7 ultramicrotome and mounted on copper grids. TEM images were acquired using a Hitachi HT7800 TEM equipped with an Eagle 4k CCD digital camera. Imaging was conducted in a double‐blind manner to ensure unbiased data collection.

### ELISA and Biochemical Assays

Serum concentrations of VC were determined using an ELISA kit (Jianglai Biotechnology, JL11730). All assays were performed according to the manufacturer's protocols. Data acquisition was performed with the i‐Mark Microplate Reader (Bio‐rad, 19684).

### Western Blot Analysis

Protein was obtained from gastrocnemius muscles using RIPA buffer (50 mm Tris, pH 7.4, 150 mM NaCl, 1% Triton X‐100, 1% sodium deoxycholate, 0.1% SDS) containing protease/phosphatase inhibitor. The protein concentration of lysates was determined using a BCA Protein Assay Kit II (Beyotime Biotechnology, P0010) and combined with 5 × SDS‐PAGE protein loading buffer (Beyotime Biotechnology, P0286). Samples were then boiled for 10 min. The prepared samples were run on a SDS‐PAGE 8–12% gel then transferred to a PVDF membrane. Ponceau S stain was performed to monitor equal protein loading between the samples. Membranes were then blocked in 5% skimmed milk in phosphate‐buffered saline (PBS) for 1 h at room temperature and further incubated with primary antibody overnight while shaking at 4 °C. The following primary antibodies were used in the current study: TET2 (Proteintech, 21207‐1‐AP, 1:500), SVCT2 (Proteintech, 27019‐1‐AP, 1:1000), PGC‐1α (Proteintech, 66369‐A‐lg, 1:4000), α‐Tubulin (Beyotime Biotechnology, AF2827), and Vinculin (Proteintech, 26520‐1‐AP, 1:4000). The next day, the membrane was washed three times with PBS‐Tween (Tween‐20, 0.1%) and incubated with anti‐rabbit/mouse horseradish peroxidase‐conjugated secondary antibody for 2 h at room temperature (Beyotime Biotechnology, A0208 and A0216, 1:10000). Proteins were visualized using enhanced chemiluminescence reagents (Cowin Biotechnology, CW0049) and exposed with a ChemiDoc Touch Imaging System (Bio‐rad, 732BR2244). Data acquisition and analysis were performed with the Image Lab software (Bio‐rad, Version 3.0) and Image J software.^[^ ^67^ ^]^

### Myogenic Cells Isolation and Cell Culture

Hindlimb skeletal muscles from 8‐week‐old wild type C57BL/6J mice were dissected and washed with 70% ethanol and PBS, then minced. Thereafter, the tissue was digested with 1.5 U mL^−1^ collagenase D, 2.4 U mL^−1^ dispase, and 2.5 mm CaCl_2_ for 60 min at 37 °C in an incubator. The digestion was terminated by adding F10 (Sigma–Aldrich, N6635) medium with 20% FBS, and the resulting cell suspension was filtered and centrifuged. The cells were then resuspended in an F10 medium supplemented with F10 medium with 20% FBS, 5 ng mL^−1^ FGF‐Basic (Gibco, PHG0026), and 0.5% chicken embryo extract (US biological, c3999) (Growth medium), and plated onto non‐Matrigel‐coated plates for 2 h to remove fibroblasts. Finally, the cell suspension was transferred to fresh Matrigel‐coated plates and cultured in growth medium or cultured in differentiation medium (F10 medium with 2% horse serum and 1% penicillin/ streptomycin).^[^ ^68^ ^]^

### Human Myogenic Cell

Human myogenic cells (HMCs) were obtained from Procell company (Procell Life Science and Technology, CP‐H095). HMCs were maintained in a DMEM medium (Gibco, C11330500BT) supplemented with FBS, EGF, and bFGF; plated onto polylysine‐coated plates; and cultured in DMEM supplemented with FBS, EGF, and bFGF (Procell Life Science and Technology, CMPH095), or cultured in differentiation medium (F10 medium with 2% horse serum and 1% penicillin/ streptomycin).

### Small Interfering RNAs

Both human (Locus ID. 54790) and mouse (Locus ID. 214133) TET2 siRNA were purchased from OriGene Technologies. Prior to siRNA transfection, human and murine myogenic cells were seeded to 60% confluence on 6‐well plates and transfected using Lipofectamine 3000 (Thermo Fisher Scientific, L3000015) and Opti‐MEM serum‐free medium (Thermo Fisher Scientific, 31985070). Following the manufacturer's protocol, Opti‐MEM serum‐free medium was used to prepare Lipofectamine 3000 and siRNA (20 nm) working solutions separately, then mixed and incubated at room temperature for 5 min before being added to the culture medium. After shaking to mix, the cells were transfected for 48 h. Subsequently, the culture medium was replaced with differentiation medium, and RNA and protein were harvested after vitamin C treatment according to the experimental design.

### Histological Analysis

For immunocytochemical (ICC) staining, the anterior tibialis muscle was embedded in astragalus gel and rapidly frozen using isopropanol precooled with liquid nitrogen. Sections (10 µm thickness) of anterior tibialis muscles were prepared for H&E (Shanghai Yuanye Biotechnology, R24044) and SDH (Solarbio, G2000) staining, followed by calculation of CSA and SDH activity using image J.

To assess muscle fiber type, transverse sections of 10 µm were fixed in 4% paraformaldehyde at room temperature for 10 min, with heat‐induced epitope retrieval (HIER) (10 mm of sodium citrate, 0.05% Tween‐20, pH 6.0, 95 °C for 10 min), and then, the samples were incubated with PBS containing 0.25% Triton X‐100 for 20 min. Afterward, the samples were incubated with 5% bovine serum albumin (BSA) and Fab fragment antibodies for 1 h to block the unspecific binding of the antibodies. Subsequently, the samples were stained overnight for anti‐myosin heavy chain (MHC) I (DSHB, BA‐D5, 1:100), anti‐MHC IIa (DSHB, SC‐71, 1:500), and anti‐MHC IIb (DSHB, BF‐F3, 1:100) mouse monoclonal antibodies at 4 °C. For the secondary antibodies, goat anti‐mouse IgG2b Alexa647 (Jackson ImmunoResearch, 115605003, 1:200), goat anti‐mouse IgG1 Alexa 594 (Jackson ImmunoResearch, 115587185, 1:200), goat anti‐mouse IgM Alexa 488 (Jackson ImmunoResearch, 115545003, 1:200), and TOM20 (CST, 42406, 1:500) antibodies were applied in the current study, DAPI was used to visualize nuclei.

Imaging was performed using a Zeiss laser confocal microscope (LSM 800, Germany) by scanning the cross‐section of the anterior tibialis muscle. The number and proportion of different MHC subtypes were quantified using Image J software.

### MethylC‐Capture Sequencing and Data Analysis

Genomic DNA was extracted from quadriceps muscle using a gDNA extraction kit from Omega Bio‐Tek Research (USA) and fragmented into 150–200 bp fragments using Covaris E‐Series instruments (Australia). The DNA library was prepared using the SureSelect Methyl‐seq Library Prep Kit from Agilent Technologies (USA), and bisulfite conversion was performed using the EZ DNA Methylation‐Gold Kit from Zymo Research (USA). The resulting DNA was desulphonated, and its quality and quantity were assessed using an Agilent 2100 Bioanalyzer. The DNA samples were sequenced on the HiSeq X ten Platform (Illumina Technologies, USA) with 150 bp paired end reads at the Shanghai Biotechnology Corporation (China).

The quality of raw data was assessed by FastQC v0.11.5, and clean data were generated using Trim galore v0.4.1. The reference genome used was Mouse MM10, and genome alignment was conducted using Bismark V0.15.0. Deduplicate Bismark was employed to remove potential PCR redundancy. Information on cytosine (C), CpG C, and 5 mC was generated through genome alignment. The 5 mC methylation levels were compared between groups and annotated using the Bioconductor package. DMSs were identified with an average methylation level difference > 10% and 5 mC with a *p*‐adjust < 0.05. DMS showing the same trend in the F1 and F2 generations were selected. Based on the annotation, methylation changes in all DMSs within the promoter‐transcription start site (TSS) were analyzed.

### Statistical Analysis

All data were statistically analyzed and graphed with GraphPad Prism 8.3 software (GraphPad Software, San Diego, CA, USA). Most experiment data were presented as the mean ± SEM for in vitro or ex vivo studies, respectively. Comparisons among multiple groups were performed using one‐way analysis of variance (ANOVA) followed by Bonferroni's post hoc test or two‐way ANOVA followed by Tukey's post hoc test, as appropriate. For correlation analysis involving four biological variables: protein expression (SVCT2, PGC‐1α) and VC concentration (plasma, skeletal muscle), Spearman correlation coefficient was calculated for each pair of variables in offspring from M‐Con or M‐Ex group at weaning. Maximal running speed data for F0, F1, F2, and F3 mice were analyzed using Kaplan–Meier survival curves, and the statistical difference was determined by the Log‐rank test. For MCC‐seq data, principal component analyses, identification of differentially expressed genes, and hierarchical cluster analyses were performed to assess global patterns. Differential methylation sites (DMSs) analysis was performed on the F1 Ex, F2 Ex group, and F1 Con group using R package methylKit, version 0.9.5, which applied Fisher's test, adjusted *p‐*value less than 0.05 to compare methylation frequencies at specific sites or regions between groups. For frequency‐based data, such as proportions of fiber types, Pearson's chi‐squared test was used to determine whether distributions differed significantly between experimental conditions. The number of samples and *p*‐values for each measurement are provided in the figures and figure legends. Statistical significance was set at *p < 0.05*.

## Conflict of Interest

The authors declare no conflict of interest.

## Author Contributions

The experimental plan was designed by H.S. and R.D. H.S., J.L., and H.Y. performed molecular, histological, and exercise performance test. H. S., F.L., T.W., Y.L., R.H., M.C., N.S.G., X.Z., and S.F. performed experiments and animal care. H.S. analyzed MCC‐Seq data. The manuscript was written by H.S., F.L., L. Y., and R.D. with help and comments from all authors.

## Supporting information



Supporting Information

Supplemental Table 1

Supplemental Table 2

Supplemental Table 3

Supplemental Table 4

Supplemental Table 5

Supplemental Table 6

Supplemental Table 7

Supplemental Table 8

## Data Availability

The data that support the findings of this study are openly available in the Sequence Read Archive of National Library of Medicine. https://www.ncbi.nlm.nih.gov/sra/PRJNA1134620, reference number PRJNA1134620.
