# Supplementary material for: Vitamin C‐Dependent Intergenerational Inheritance of Enhanced Endurance Performance Following Maternal Exercise
Source: Adv Sci (Weinh). 2025 Feb 8;12(13):2408912. doi: 10.1002/advs.202408912 (PMC11967756; doi:10.1002/advs.202408912)
Supplement: Supplementary file 1 — Supporting Information [file ADVS-12-2408912-s002.docx]

Supplementary Materials for

**Vitamin C-Dependent Intergenerational Inheritance of Enhanced Endurance Performance Following Maternal Exercise**

Haiwang Shi *et al.*

*Corresponding author. Email: duanrui@m.scnu.edu.cn

**This PDF file includes:**

Supplementary Materials: Figures. S1 to S15


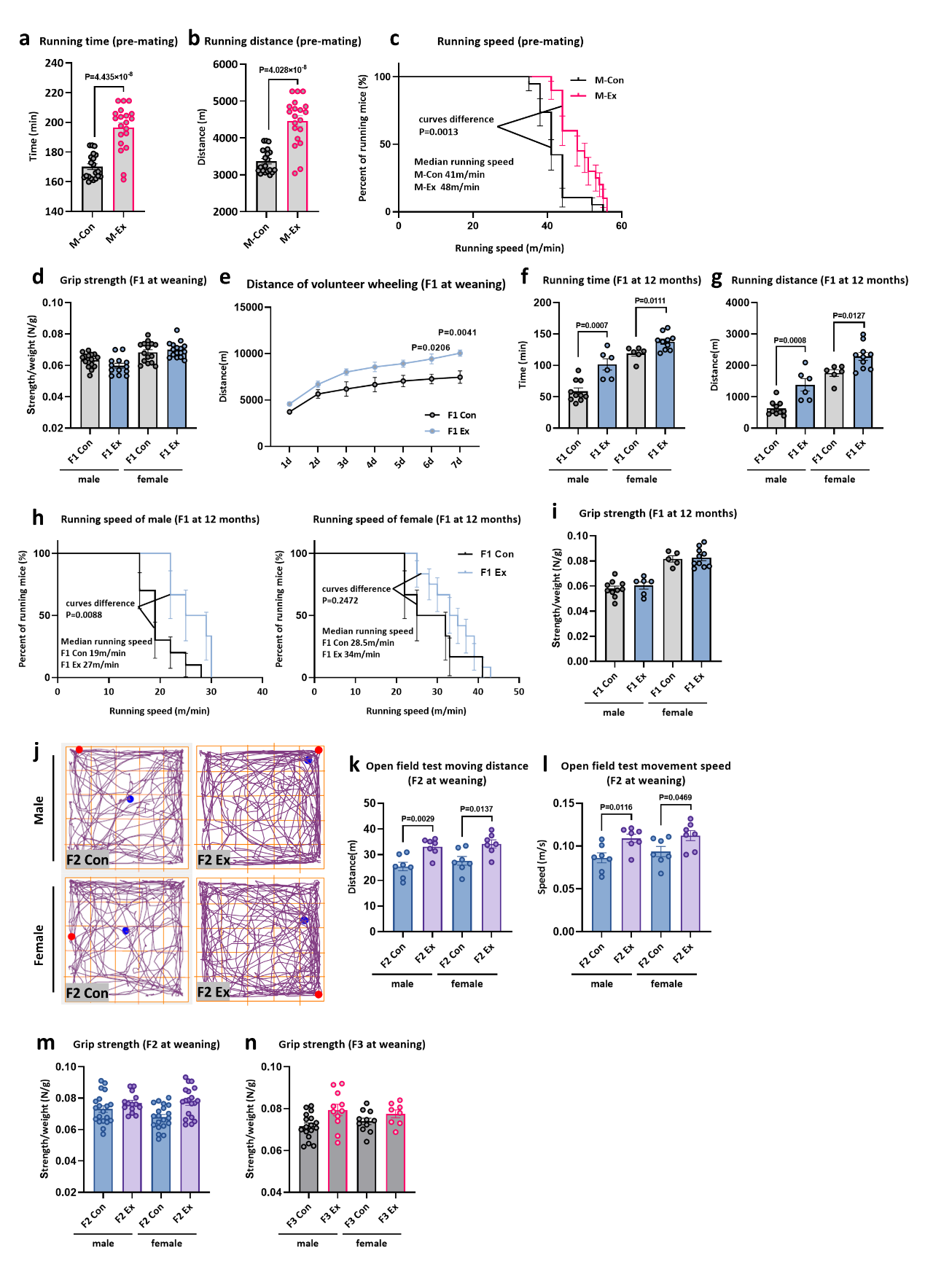
 **Extended descriptions of results.**

**Fig. S1**

**a-c.** Exercise performance test after four weeks of treadmill exercise training. Running time (a), distance (b), and population plotted against running speed to exhaustion (c) in female mice (n=20). **d.** Forelimb maximum grip strengths of F1 generation mice were measured 5 times successively and normalized against body mass (Male: F1 Con n=16, F1 Ex n=13. Female: F1 Con n=16, F1 Ex n=16). **e.** The distance of free wheel running in F1 mice within one week (n=10). **f-h.** Treadmill endurance performance of F1 generation mice at 12 months. Running time (f), distance (g), and population plotted against running speed to exhaustion (h) in F1 at 12 months (Male: F1 Con n=10, F1 Ex n=6. Female: F1 Con n=6, F1 Ex n=10). **i.** Forelimb maximum grip strengths of F1 at 12 months. **j-l**. An illustrative example of the travel pathway of mice in open field testing, with a red dot representing the start point and blue representing the stop point (j), moving distance (k) and movement speed (l) (n=7). **m, n**. Forelimb maximum grip strengths of F2 (m) and F3 (n) at weaning. Statistical analyses were performed using t-test (a, b, d, f, g, i, k-n), two-way ANOVA with Tukey’s post hoc analysis (e) and Log-rank test (c, h). Data presented as mean ± SEM.


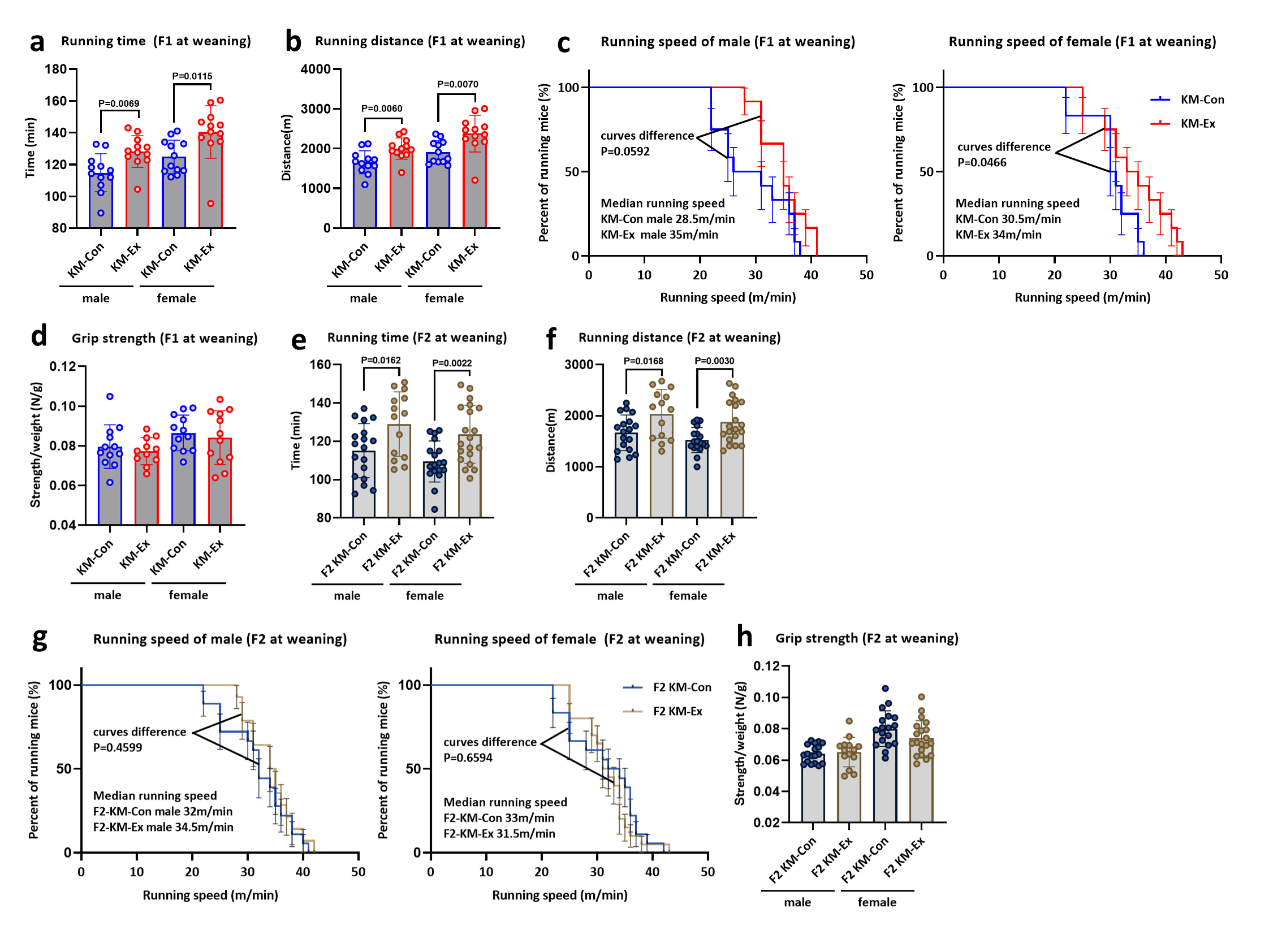
**Fig. S2**

Kunming mice were randomly divided into a control group (KM-Con) and an exercise group (KM-Ex). The KM-Ex group underwent 4 weeks of treadmill exercise, while the KM-Con group remained sedentary. After this period, both groups mated with age-matched, non-trained wild-type (WT) mice to generate F1 and F2 offspring. **a-c.** Results of exercise performance test in F1 Kunming offspring. Running time (a), distance (b), and the population plotted against running speed to exhaustion (c) in F1 generation mice. **d.** Forelimb maximum grip strengths of F1 generation mice were measured 5 times successively and normalized against body mass (n=12 per group for panels a-d). **e-g.** Results of exercise performance test in F2 Kunming offspring. Running time (e), distance (f), and population plotted against running speed to exhaustion (g) in F2 generation mice (Male: F2 Con n=18, F2 Ex n=14. Female: F2 Con n=18, F2 Ex n=20 for panels e-h). **h.** Forelimb maximum grip strengths of F2 generation mice were measured 5 times successively and normalized against body mass. Statistical analyses were performed using t-test (a, b, d, e, f, h) and Log-rank test (c, g). Data presented as mean ± SEM.


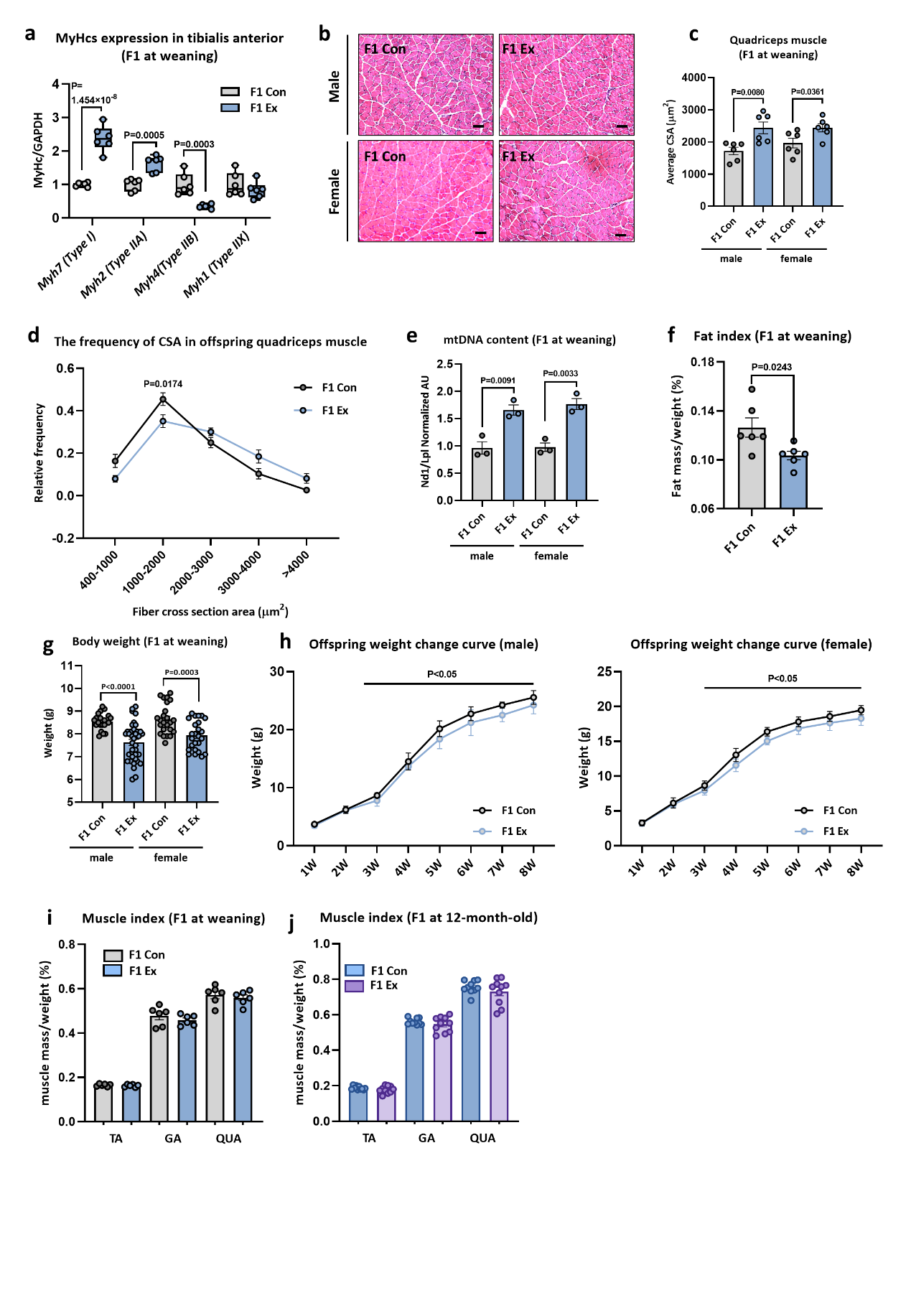
**Fig. S3**

**a.** The gene expression of *MyHc* isoforms in skeletal muscle of the F1 generation at weaning (n=6). **b**. **c.** Representative H&E staining (b, the scale bar represents 100 μm) and the statistical analysis of average cross-section area (c) (n=6). **d**. Distribution of muscle fiber sizes in F1 generation skeletal muscle (n=6). **e**. Mitochondrial DNA (mtDNA, Ndl) was quantified by qPCR using nuclear DNA (gDNA, Lpl) as a standard (n=3). **f, g.** Effects of maternal exercise on inguinal fat index (f, n=6) and body weight (g, Male: F1 Con n=21, F1 Ex n=24. Female: F1 Con n=24, F1 Ex n=25;) in offspring mice. **h.** Effects of maternal exercise on weight gain during development in F1 offspring mice (Before weaning, Male: F1 Con n=19, F1 Ex n=16. Female: F1 Con n=18, F1 Ex n=19; After weaning, Male: F1 Con n=16, F1 Ex n=13. Female: F1 Con n=15, F1 Ex n=16). **i, j.** The skeletal muscle weight of F1 generation mice was normalized to body weight both at weaning (k, n=6) and at 12 months of age (l, n=10). Statistical analyses were performed using t-test (a, c, e, f, g, i, j) and two-way
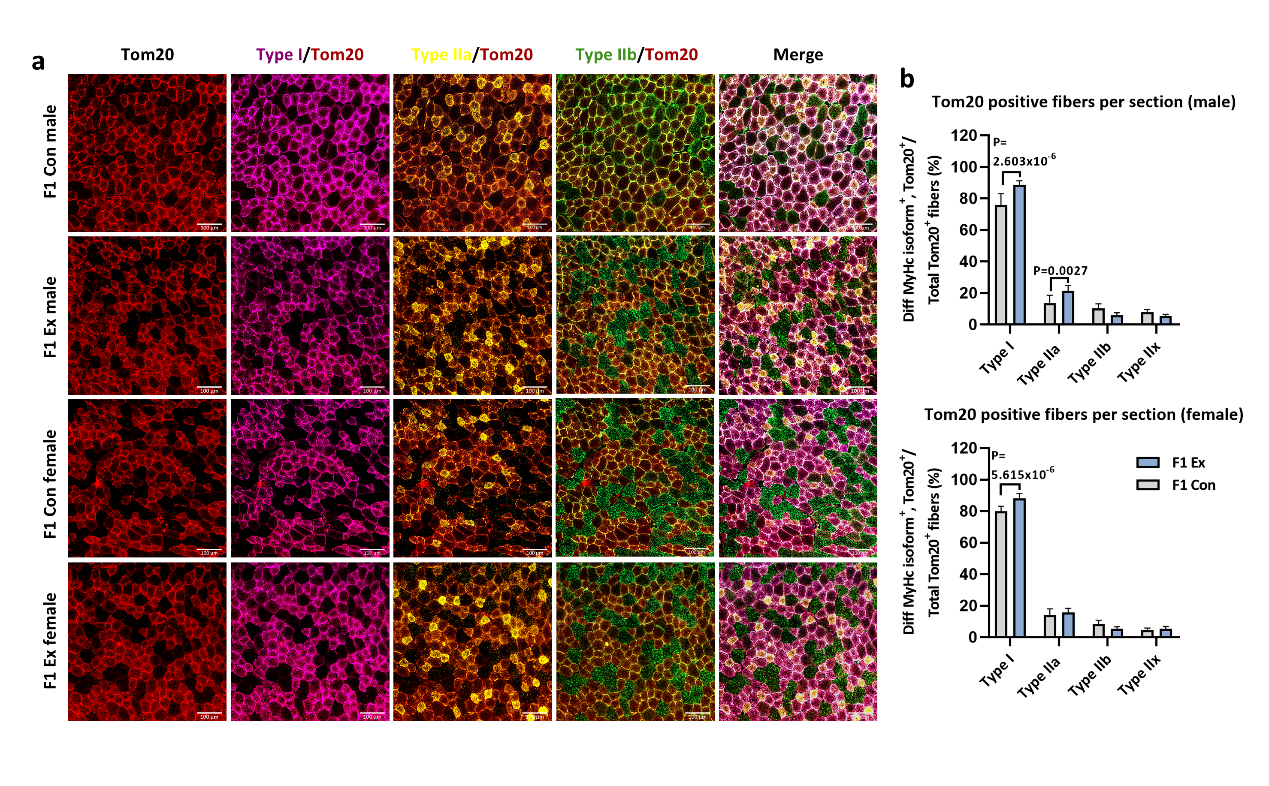
ANOVA with Tukey’s post hoc analysis (d, h). Data presented as mean ± SEM.

**Fig. S4**

a. Representative immunofluorescence staining images of the mitochondrial membrane protein marker TOM20 with specific *MyHC* isoforms in the tibial anterior muscle of F1 generation at weaning, the scale bar represents 100 μm (n=3). **b.** Quantitative analysis of Tom20 positive fiber percentage in different fiber types of offspring muscle (n=3). Statistical analyses were performed using two-way ANOVA with Tukey’s post hoc analysis (b). Data presented as mean ± SEM.


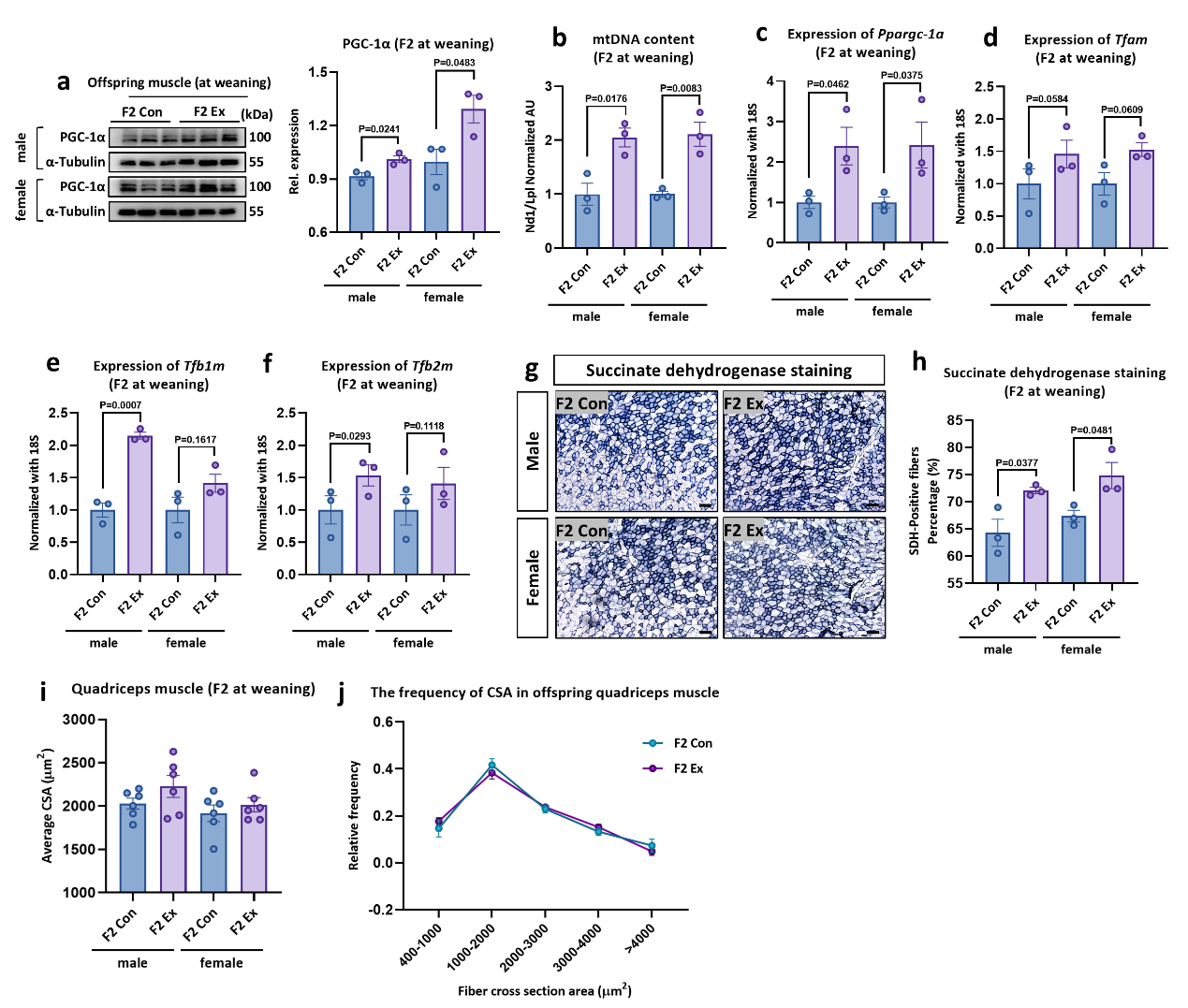
**Fig. S5**

**a.** Representative images of Western blot analysis of PGC-1α in gastrocnemius muscles of F2 generation (n=3). **b-f.** Analysis of genes related to mitochondrial biogenesis and mitochondrial copy number in F2 generation at weaning (n=3). **g, h.** Representative succinate dehydrogenase (SDH) staining (g) and quantification (h) of SDH-positive fibers in F2 generation (n=3). The scale bar represents 100 μm. **i, j.** The average cross-sectional area (i) and distribution of muscle fiber sizes (j) in F2 generation skeletal muscle (n=6). Statistical analyses were performed using t-test (a-f and h, i) and two-way ANOVA with Tukey’s post hoc analysis (j). Data presented as mean ± SEM.


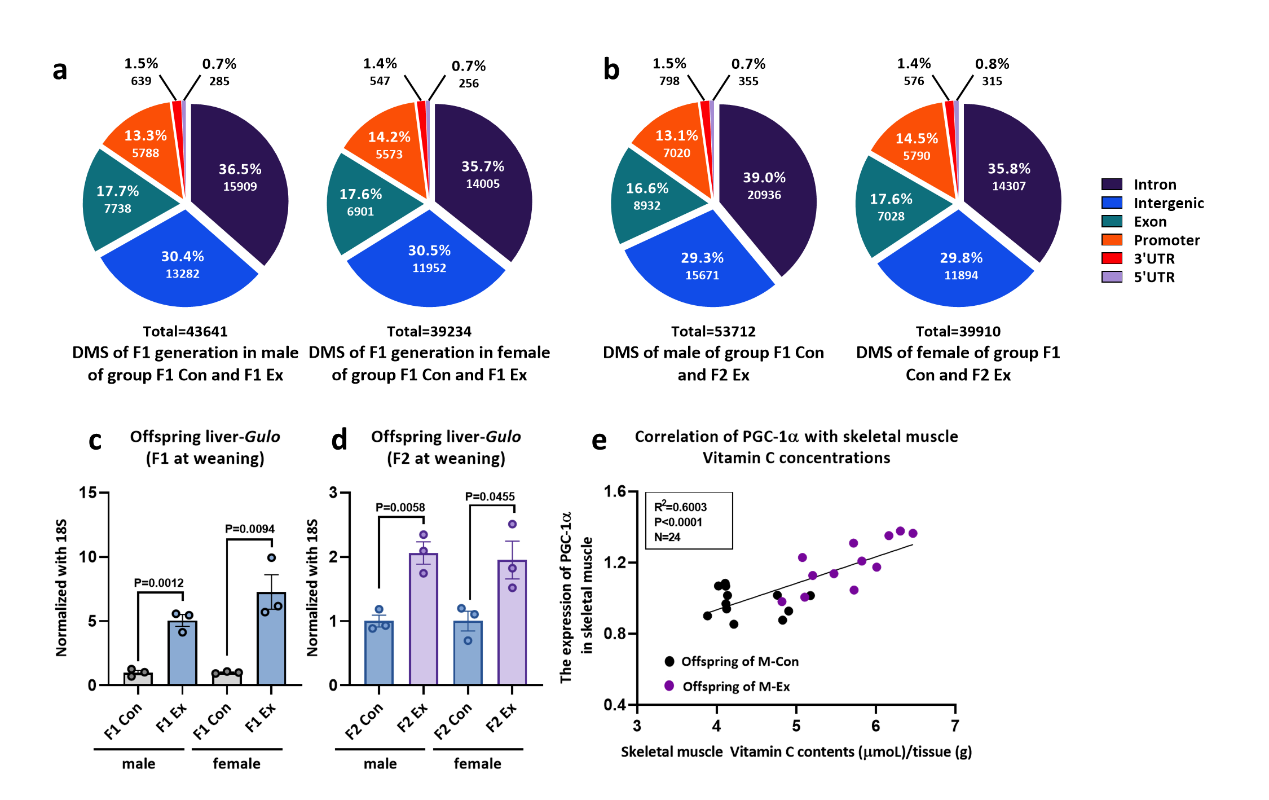
**Fig. S6**

**a, b.** The distribution of differential methylation sites in different gene elements, F1 Con vs. F1 Ex (a), and F1 Con vs. F2 Ex (b) (n=3). **c, d**. The expression of *Gulo* in liver of F1 (e) and F2 (f) generation mice at weaning (n=3). **e**. Pearson correlation coefficient between VC concentration and PGC-1α protein expression in skeletal muscle of F1 and F2 generations in the M-Con group (black) and M-Ex group (purple) at weaning (n=24). Statistical analyses were performed using t-test (c, d). Data presented as mean ± SEM.


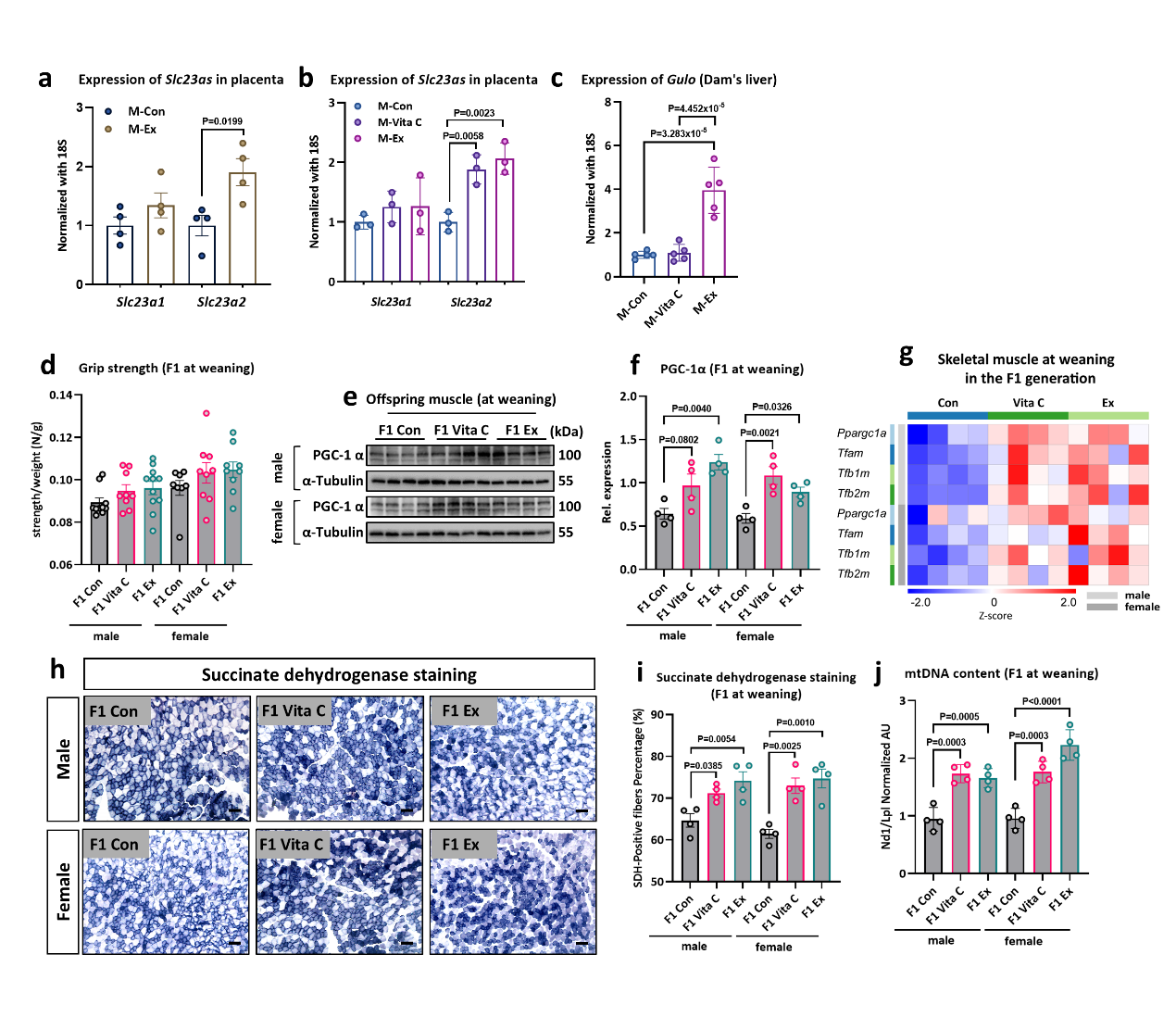
**Fig. S7**

**a**. The expression of *Slc23a1/2* in placenta at e18.5 after maternal exercise (n=4). **b**. The expression of *Slc23a1/2* in placenta at e18.5 following maternal exogenous VC supplementation during pregnancy (n=3). **c**. The expression of *Gulo* in dam’s liver following maternal exogenous VC supplementation during pregnancy (n=5). **d**. Forelimb maximum grip strengths of F1 generation mice were measured 5 times successively and normalized against body mass (Male: F1 Con n=10, F1 Vita C n=9, F1 Ex n=11. Female: F1 Con n=8, F1 Vita C n=9, F1 Ex n=9). **e, f.** Representative images of Western blot analysis of PGC-1α in skeletal muscles of F1 generation (n=4). **g.** Heatmap visualizing expression of genes related to mitochondrial biogenesis in F1 generation at weaning (n=4). **h, i.** Representative succinate dehydrogenase (SDH) staining (h) and quantification (i) of SDH-positive fibers (n=4). The scale bar represents 100 μm. **j**. Mitochondrial DNA (mtDNA, *Ndl*) was quantified by qPCR using nuclear DNA (gDNA, *Lpl*) as a standard (n=4). Statistical analyses were performed using t-test (a) and one-way ANOVA with Bonferroni’s post hoc analysis (b-d, f, g and i, j). Data presented as mean ± SEM.


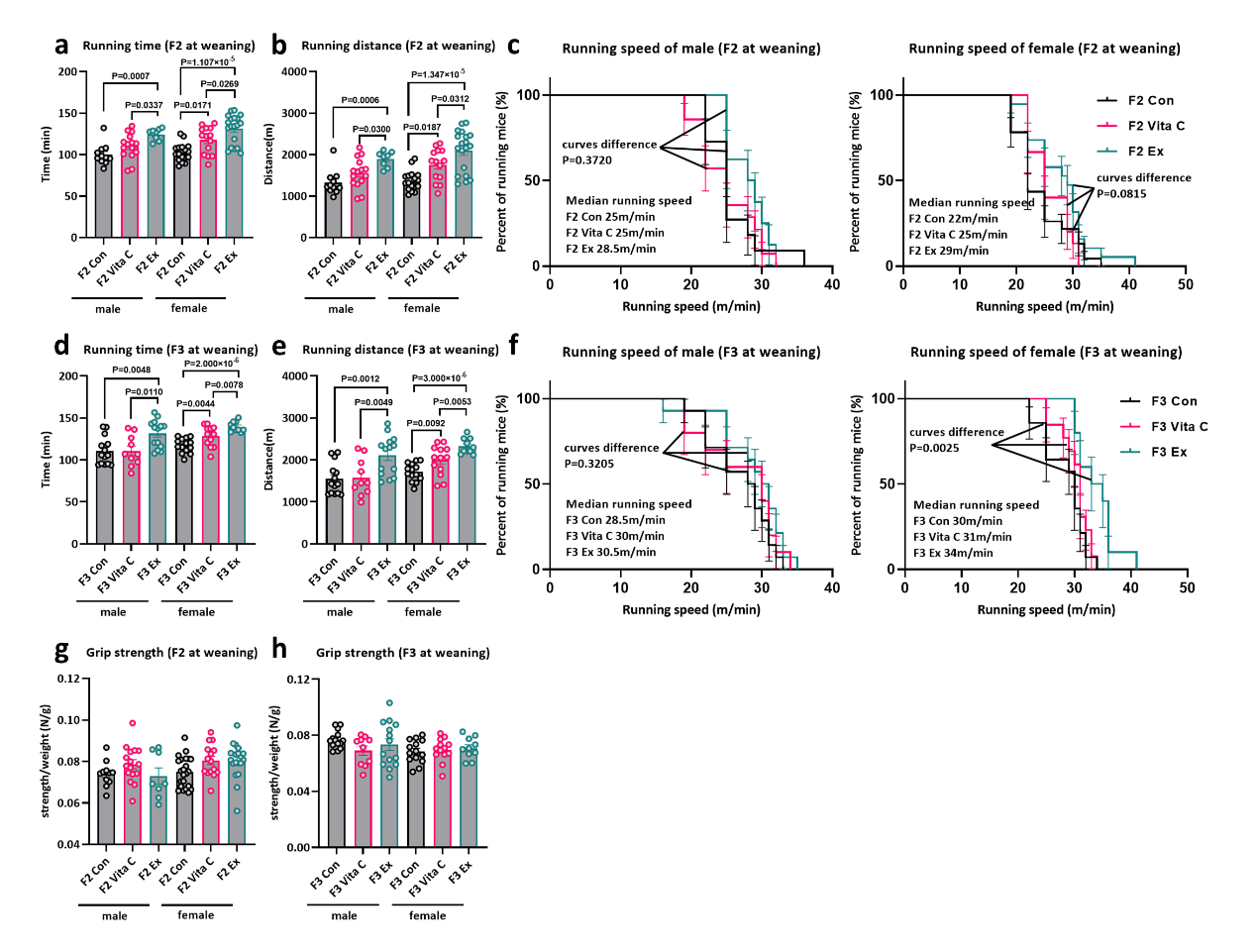


**Fig. S8**

**a**-c**.** The effect of maternal exogenous VC supplementation before and during pregnancy on the endurance performance of F2 mice. Running time (a), distance (b), and population plotted against running speed to exhaustion (c) (Male: F2 Con n=11, F2 Vita C n=15, F2 Ex n=8. Female: F2 Con n=17, F2 Vita C n=14, F2 Ex n=19). **d-f.** The effect of maternal exogenous VC supplementation before and during pregnancy on the endurance performance of F3 generation. Running time (d), distance (e), and population plotted against running speed to exhaustion (f) (Male: F3 Con n=14, F3 Vita C n=10, F3 Ex n=14. Female: F3 Con n=14, F3 Vita C n=13, F3 Ex n=10). **g, h.** Forelimb maximum grip strengths of F2 (g) and F3 (h) generation mice were measured 5 times successively and normalized against body mass. Statistical analyses were performed using one-way ANOVA with Bonferroni’s post hoc analysis (a, b, d, e, g) and the Log-rank test (c, f). Data presented as mean ± SEM


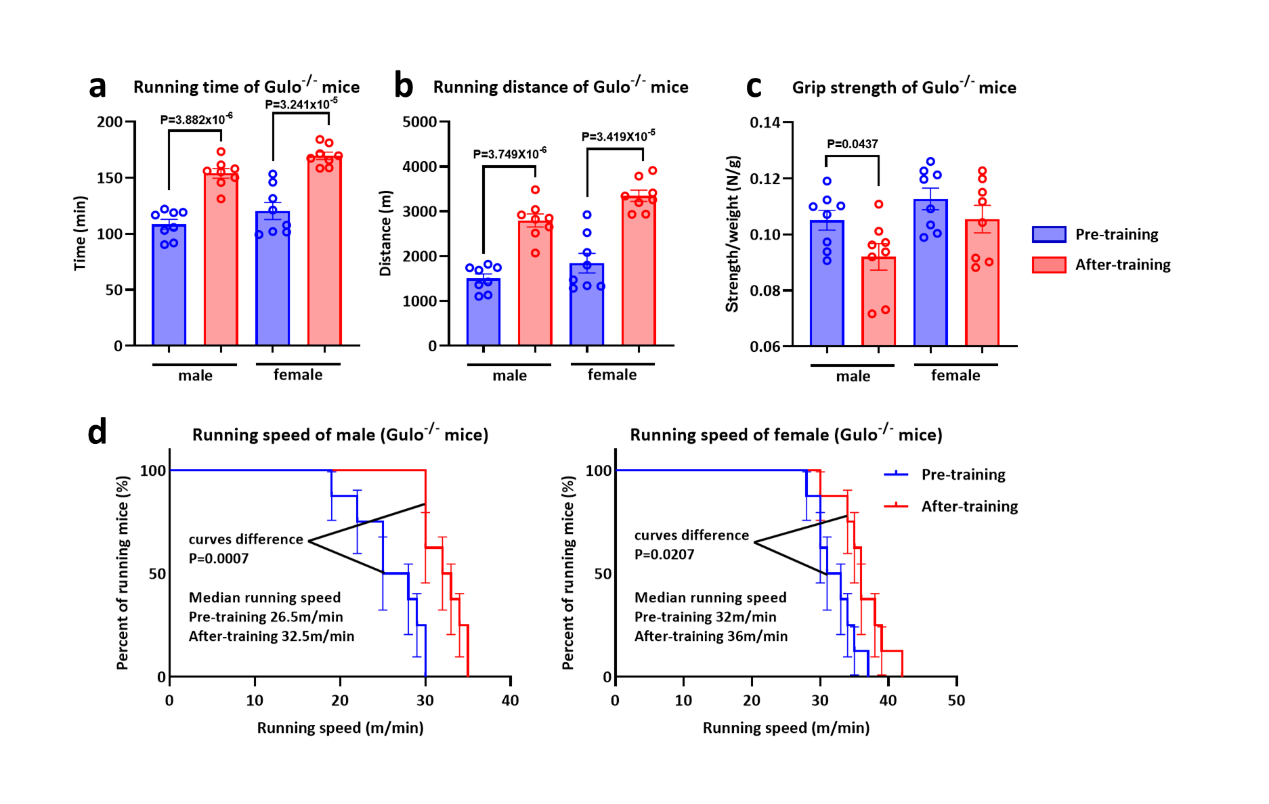
**Fig. S9**

The effect of 4 weeks treadmill exercise on the endurance performance of *Gulo^-/-^* mice. Running time (a), distance (b), forelimb maximum grip strengths (c) and population plotted against running speed to exhaustion (d) (Male: pre-training n=8, after-training n=8. Female: pre-training n=8, after-training n=8). Statistical analyses were performed using t-test (a-c) and the Log-rank test (d). Data presented as mean ± SEM.


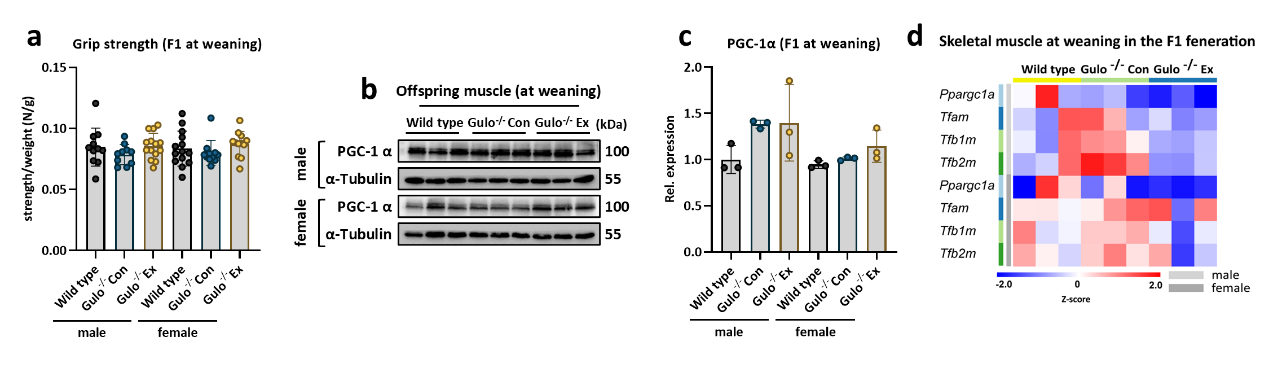
**Fig. S10**

**a.** Forelimb maximum grip strengths of offspring from *Gulo*^-/-^ mice were measured 5 times successively and normalized against body mass (Male: Wild type n=11, *Gulo*^-/-^ Con n=10, *Gulo*^-/-^ Ex n=14. Female: Wild type n=14, *Gulo*^-/-^ Con n=10, *Gulo*^-/-^ Ex n=10). **b, c.** Representative images of Western blot analysis of PGC-1α in skeletal muscle of offspring at weaning after maternal exercise in *Gulo*^-/-^ mice (n=3). **d.** Analysis of genes related to mitochondrial biogenesis in offspring at weaning after maternal exercise in *Gulo*^-/-^ mice (n=3). Statistical analyses were performed using one-way ANOVA with Bonferroni’s post hoc analysis (a-d). Data presented as mean ± SEM.


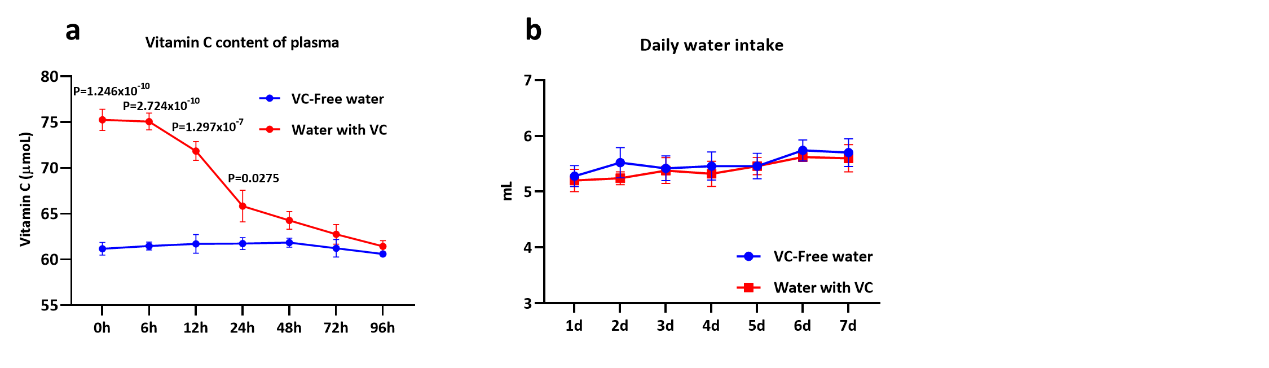


**Fig. S11**

**a.** The change curve of plasma VC content during the VC withdrawal period after 4 weeks of exogenous VC supplementation (n=3). **b.** The curve of daily water intake in mice (n=5). Statistical analyses were performed using two-way ANOVA with Tukey’s post hoc analysis (a, b). Data presented as mean ± SEM.


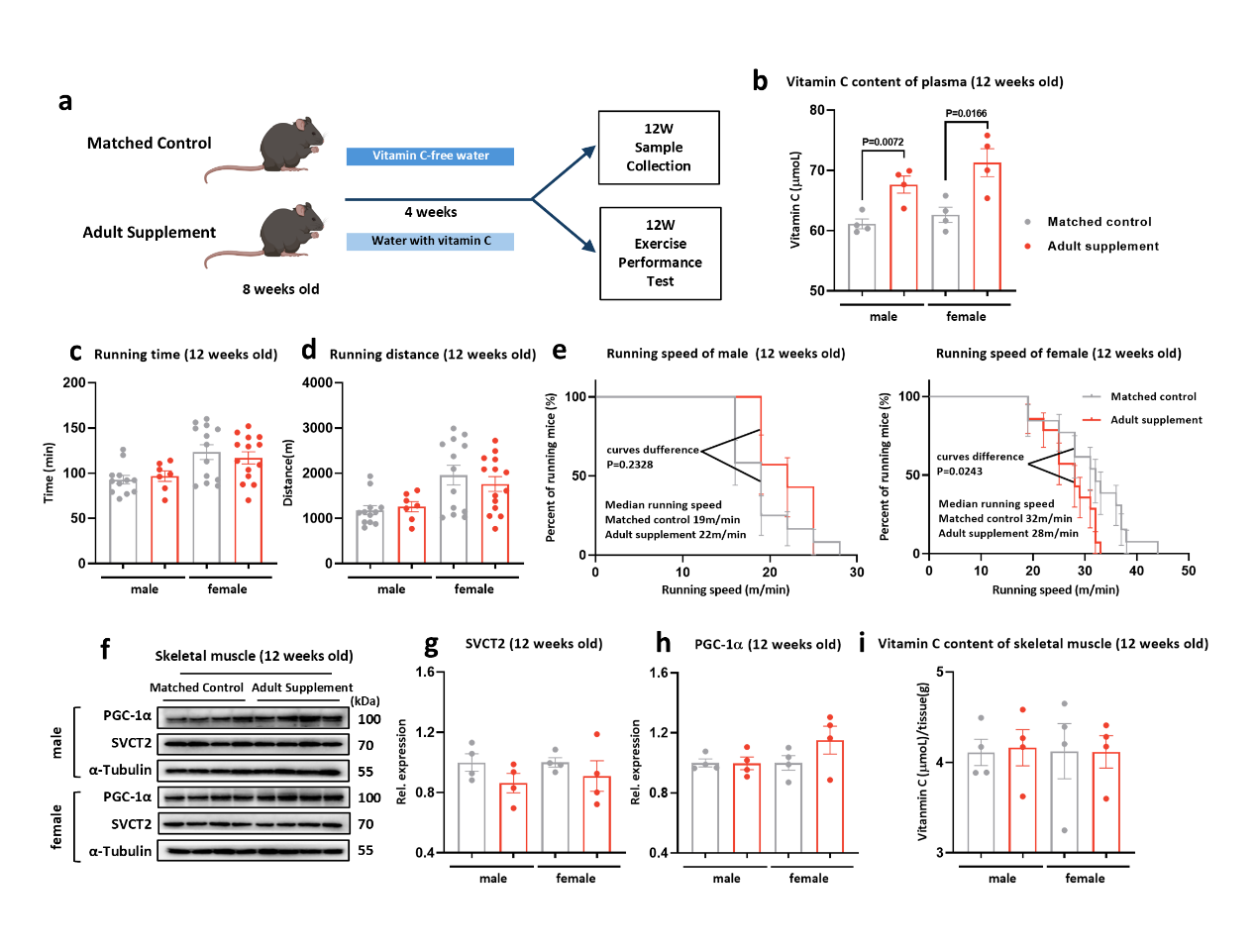
**Fig. S12**

**a**. A schematic diagram of the experiment. The wild-type mice were supplemented exogenously with VC by drinking water for 9-12 weeks age. **b.** Plasma vitamin C concentrations in mice supplemented with VC at 9-12 weeks of age (n=4). **c-e**. Exercise capacity testing of exogenous VC supplementation before or after weaning. Running time (l), distance (m), and population plotted against running speed to exhaustion (n) (Male: Matched control n=12, adult supplement n=7. Female: Matched control n=13, adult supplement n=14). **f-h**. Representative images of Western blot analysis of SVCT2 (g) and PGC-1α (h) in skeletal muscle of mice supplemented with VC at 9-12 weeks of age (n=4). **i**. Skeletal muscle VC concentrations in mice supplemented with VC at 9-12 weeks of age (n=4). Statistical analyses were performed using t-test (b, c, d, g, h, i) and the Log-rank test (e). Data presented as mean ± SEM.

**Fig. S1**
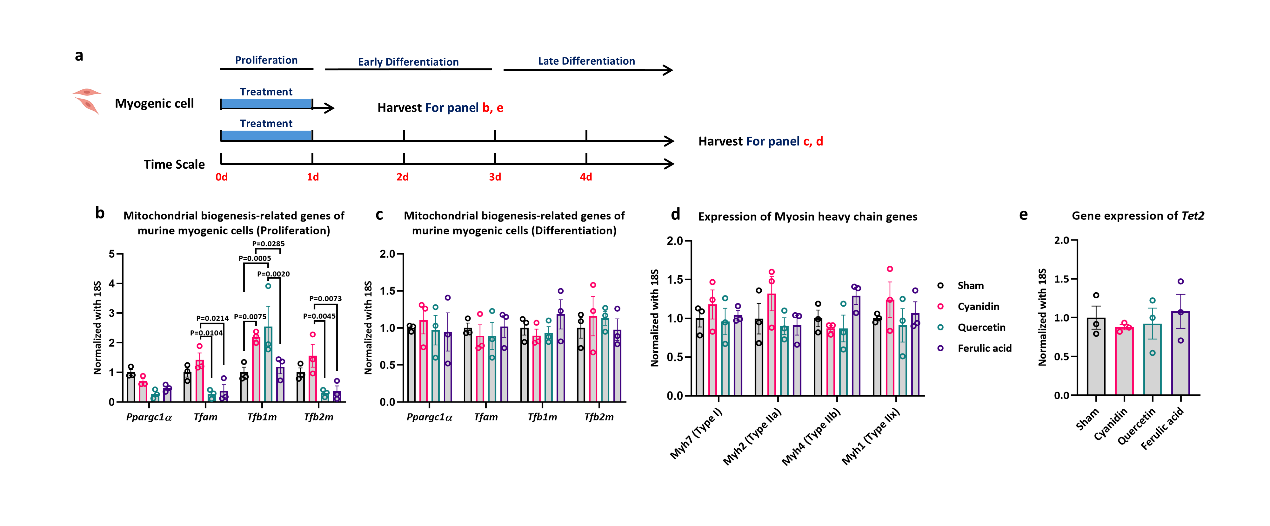
**3**

**a.** Schematic showing exogenous *Cyanidin, Quercetin* and *Ferulic acid* supplementation during murine myogenic cell proliferation respectively, and cell samples were collected during both the proliferation stage and the differentiation stage. **b, c.** The effects of antioxidant treatment on the gene expression associated with mitochondrial biogenesis in murine myogenic cell at the proliferative stage (b) and the differentiated (c) stage respectively (n=3). **d.** The effects of antioxidant treatment on the gene expression of different *MyHc* isoforms in murine myogenic cells (n=3). **e.** The effects of antioxidant treatment on the gene expression of *Tet2* in murine myogenic cells. Statistical analyses were performed using one-way ANOVA with Bonferroni’s post hoc analysis (b-e). Data presented as mean ± SEM.


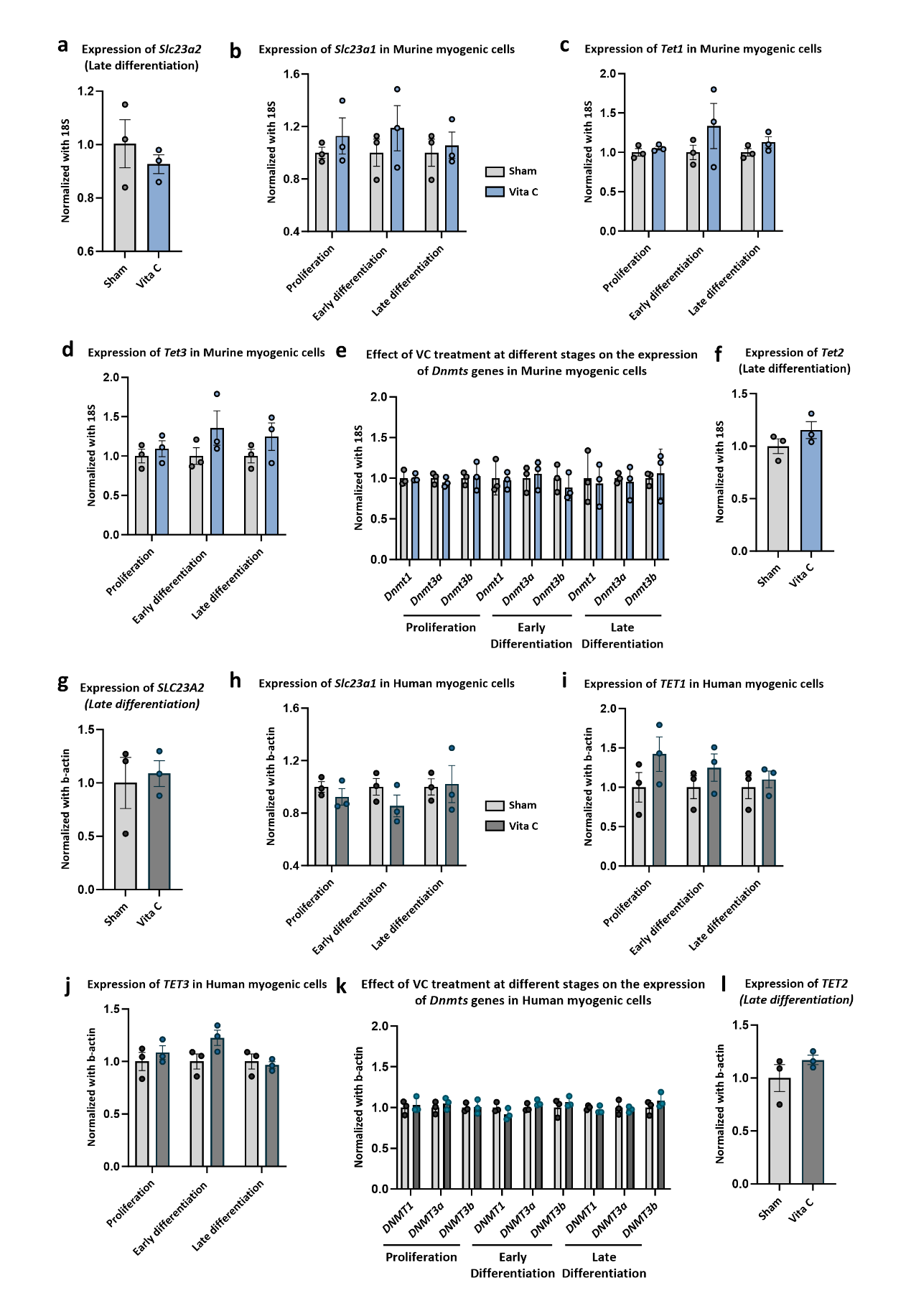
**Fig. S14**

**a, g.** Effect of exogenous VC treatment on *Slc23a2/SLC23A2* expression in murine (a) and human (g) myogenous cells at the late stage of differentiation (n=3). **b, h.** Effect of exogenous VC treatment on *Slc23a1/SLC23A1* expression in murine (b) and human (h) myogenous cells during myogenic cell proliferation, early and late differentiation (n=3). **c, d and i, j**. Genes expression of *Tet1/TET1* (c, i) and *Tet3/TET3* (d, j) were detected in murine (c, d) and human (i, j) myogenic cells supplemented with VC during proliferation, early differentiation and late differentiation (n=3). **e, k.** Effect of exogenous VC treatment on *Dnmts/DNMTs* expression in murine (e) and human (k) myogenous cells during myogenic cell proliferation, early and late differentiation (n=3). **f, l.** Genes expression of *Tet2/TET2* in murine (f) and human (l) myogenic cells at the late stage of differentiation (n=3). Statistical analyses were performed using t-test (a-l). Data presented as mean ± SEM.


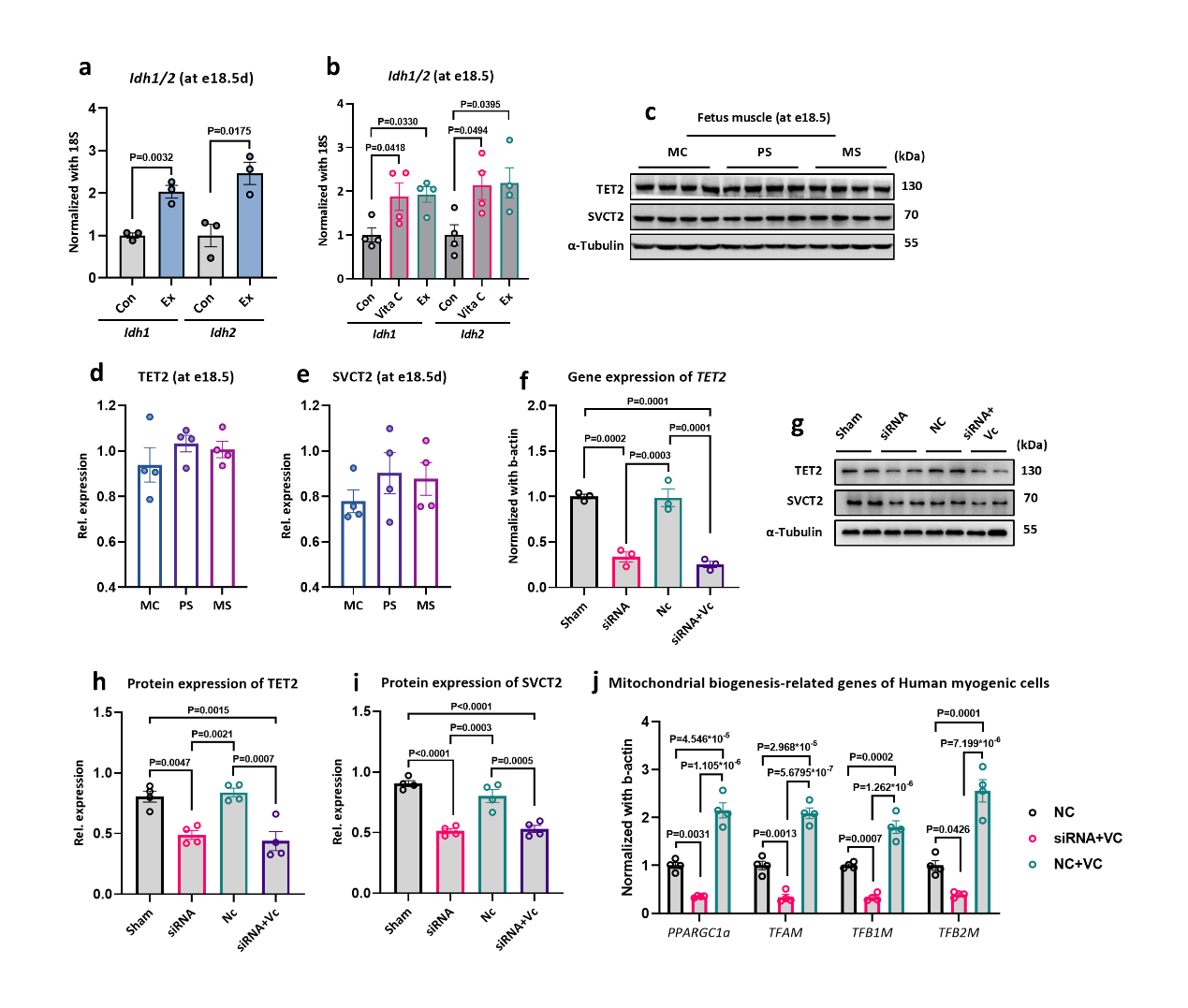
**Fig. S15**

**a, b**. Expression of *Idh1/2* in fetal skeletal muscle at e18.5 after maternal exercise (a) (n=3) or exogenous VC supplementation (b) (n=4) during pregnancy. **c-e.** Representative images of Western blot analysis of TET2 (d) and SVCT2 (e) in fetal skeletal muscle of MC, PS and MS dams (n=4). **f**. The gene expression of *TET2* after siRNA transfection of human myogenic cells (n=3). **g-i**. Representative images of Western blot analysis of TET2 (h) and SVCT2 (i) after siRNA transfection of human myogenic cells (n=4). **j**. The gene expression of related to mitochondrial biogenesis after siRNA transfection of human myogenic cells (n=3). Statistical analyses were performed using t-test (a) and one-way ANOVA with Bonferroni’s post hoc analysis (b-j). Data presented as mean ± SEM.
